# Reading bots: The implication of deep learning on guided reading

**DOI:** 10.3389/fpsyg.2023.980523

**Published:** 2023-02-06

**Authors:** Baorong Huang, Juhua Dou, Hai Zhao

**Affiliations:** ^1^Institute of Corpus Studies and Applications, Shanghai International Studies University, Shanghai, China; ^2^School of International Cooperation, Guangdong Polytechnic of Science and Technology, Guangzhou, China; ^3^Unikl Business School, University of Kuala Lumpur, Kuala Lumpur, Malaysia; ^4^Department of Computer Science and Engineering, School of Electronic Information and Electrical Engineering, Shanghai Jiao Tong University, Shanghai, China

**Keywords:** deep learning, reading comprehension, technology, natural language processing, reading stages

## Abstract

This study introduces the application of deep-learning technologies in automatically generating guidance for independent reading. The study explores and demonstrates how to incorporate the latest advances in deep-learning-based natural language processing technologies in the three reading stages, namely, the pre-reading stage, the while-reading stage, and the post-reading stage. As a result, the novel design and implementation of a prototype system based on deep learning technologies are presented. This system includes connections to prior knowledge with knowledge graphs and summary-based question generation, the breakdown of complex sentences with text simplification, and the auto-grading of readers' writing regarding their comprehension of the reading materials. Experiments on word sense disambiguation, named entity recognition and question generation with real-world materials in the prototype system show that the selected deep learning models on these tasks obtain favorable results, but there are still errors to be overcome before their direct usage in real-world applications. Based on the experiment results and the reported performance of the deep learning models on reading-related tasks, the study reveals the challenges and limitations of deep learning technologies, such as inadequate performance, domain transfer issues, and low explain ability, for future improvement.

## 1. Introduction

Reading comprehension is one of the primary ways for a human to acquire knowledge, and the cultivation of reading skills in students by instructors to facilitate the distillation of knowledge remains one of the central tasks in literary education. To maximize the effects of reading comprehension, instructors have developed a lot of strategies and tools, including computer technology. Computer technology is a widely used vehicle to promote literacy of students in reading, as evidenced by a large number of studies that focused on the effects of intelligent tutoring system (ITS) in the age groups of children in grades 1–3 (Hauptmann et al., 1994), kindergarten (Voogt and McKenney, 2007), K-12 (from kindergarten to 12th grade) students (Proudfoot, 2016; Xu et al., 2019; Pahamzah et al., 2022), and adults (Ramachandran and Stottler, 2000). These ITS systems assisted readers by acting as coaches (Hauptmann et al., 1994), reading companions (Madnani et al., 2019), or using augmented reality (Voogt and McKenney, 2007) to build interactive digital environments. Artificial intelligence, including Bayesian networks and fuzzy logic, was used to adaptively support students in learning environments, which had shown positive results (Eryilmaz and Adabashi, 2020).

With the growing demand for personalized tutoring, the traditional computer technology or the ITS systems that heavily rely on manually compiled reading materials, supporting quizzes, and pictures are not sufficiently flexible and expandable to cope with the massive online materials. In the era where the “digital twin” in the metaverse gradually emerges as the substitution of the real world for humans and the advances in artificial intelligence, digital text is growing at an unprecedented pace, which brings about huge challenges for instructors. To solve the problem of increasing customer interactions, using deep-learning technologies, more and more chatbots are being deployed to imitate human communication and serve customers in the service industry. However, despite the progress in natural language processing and heated waves in the commercialization of progress in chatbots, commodity recommendations, and other fields, the application of this progress for reading comprehension tutoring is still in its infancy.

In this study, we propose the concept of reading bots, which pioneers the application of the recent advances in deep learning-based natural language processing in the instructions of reading comprehension. The reading bot can act as an instructor for readers with reading difficulties or assist them in preparing for a language test. It guides the readers through reading activities in reading comprehension, including guiding questions, vocabulary building, analysis of complex and long sentences, multiple-choice question quizzes, and writing tasks. In addition, it can also assist the instructor to prepare the reading course materials with automatically generated questions, image and audio resources retrieved from knowledge graphs, and automatic grading of the essays submitted by readers.

The structure of this study is as follows: after this introduction, we review some of the studies in the reading models, the reading stages, the reading objectives, and the computer technology used in assisting reading, and then, we brief the recent developments in natural language processing. We ground the application of the recent developments with the reading rope theory that emphasizes the combination of all necessary skills for deep understanding. The “Reading-related technologies in the age of deep learning” section explains the concrete technologies that can be applied in guided reading, including word sense disambiguation, named entity recognition, knowledge graphs, question generation, text simplification, automatic short answer grading, and automatic essay scoring. In the “Model mapping and implementation for reading bots” section, we describe the design and implementation of reading bots that apply the aforementioned technologies. In the “Case studies” section, we evaluate the proposed reading bots with 10 articles from the website of the British Council^1^ (a public corporation that helps English learners) and present the performance of the deep-learning technologies, detailing their strengths and weaknesses. Finally, we point out the challenges and limitations of the current design, considering the possibilities for future research.

In this study, we aim at answering the following questions:

1) What specific technologies boosted by deep learning can be used for guided reading?
2) How do we design a reading bot that incorporates the advances in deep learning?
3) What is the performance of the current deep learning models in handling reading materials outside the predefined datasets?

## 2. Literature review: Reading models and computer technology in reading

### 2.1. Reading models revisited

Reading comprehension is the process that relates aspects of the world around us—including what we read—to the knowledge, intentions, and expectations we already have in our heads with continuous predictions based on prior knowledge (Smith, 2004). There are different models for describing reading processes, such as the simple view of reading (Gough and Tunmer, 1986), the construction-integration model (Kintsch, 1988), the reading rope (Scarborough, 2001), and Seidenberg's triangle model (Seidenberg, 2017). The simple view of reading states that both word decoding and linguistic comprehension are vital to reading comprehension. According to the construction-integration model, comprehension is the result of two core processes, namely, construction and integration. The former process activates the information from the text and prior knowledge that resides in the memory of the reader, and the latter process spreads the activation throughout this network until activation settles (Butterfuss et al., 2020). The reading rope model states that skilled reading is the combination of word recognition skills and language comprehension skills, including background knowledge, vocabulary, language structure, verbal reasoning, and literacy knowledge. Seidenberg's triangle model points out that people have to create links in reading from print to existing knowledge of the spoken language and from phonology to semantics (the meaning).

Connectivism is a new theory that emphasizes the knowledge gained through group activities. Connectivism, as defined by Siemens (Siemens, 2005), marks “tectonic shifts in society where learning is no longer an internal, individualistic activity”, and three of its core principles are as follows:

1. Learning is a process of connecting specialized nodes or information sources.
2. Nurturing and maintaining connections is needed to facilitate continual learning.
3. The ability to see connections between fields, ideas, and concepts is a core skill.

In addition, personal knowledge is comprised of a network and interacts with the knowledge of organizations or institutes in complementary circles, which allows learners to remain current in their field through the connections they have formed.

### 2.2. Reading stages and objectives

The three-stage reading process, i.e., pre-reading (into), while-reading (through), and post-reading (beyond), in consideration of three types of cultural and content schemata, text-processing schemata, and linguistic and grammatical schemata (Diaz-Rico, 2013, p. 172–179), is widely used in organizing activities in the teaching of reading (literacy). In the pre-reading stage, readers are prepared with key glossaries, pictures, background knowledge, domain-specific knowledge, or a summary of the text to arouse the prior knowledge and be ready to make connections for the assimilation of new knowledge contained in the text. In the while-reading stage, the tactics for enhancing linguistic and grammatical knowledge can be used to merge the knowledge in the text into the existing schemata of the readers. In the post-reading stage, various activities can be organized to assist readers in evaluating comprehensions, such as follow-up hard questions, summarization, purpose reflections, and reciprocal teaching.

**Figure 1:**
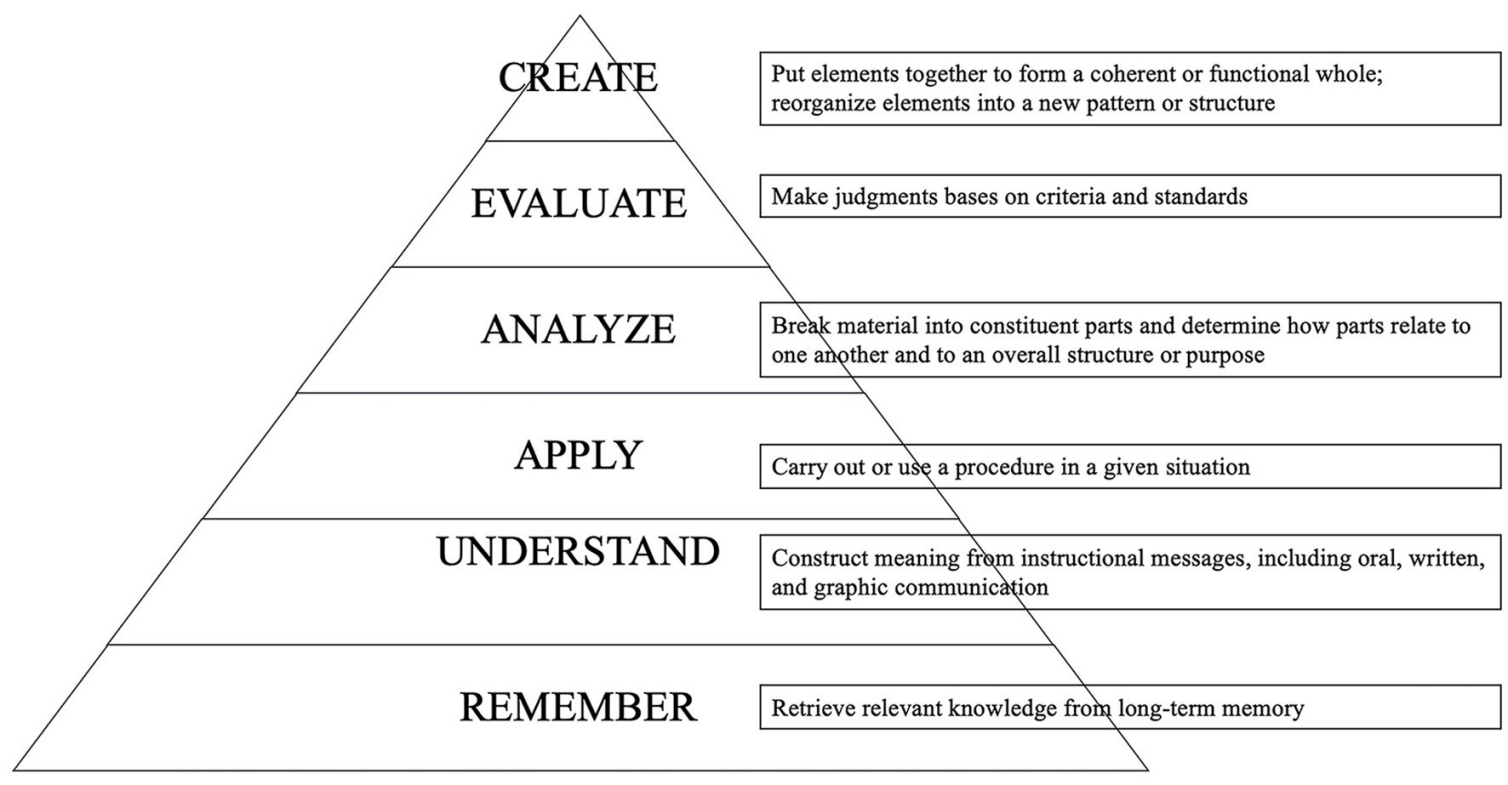
Bloom's taxonomy in cognitive processes.

The activities in the three reading stages should all contribute to and work concertedly to prepare readers for higher levels in the common educational objectives. The educational objectives, according to Bloom, can be divided into six categories as follows: knowledge, comprehension, application, analysis, synthesis, and evaluation (Bloom, 1956). In 2000, the researchers (Anderson et al., 2001, p. 21–22) revised the taxonomy and connected six new categories with the cognitive processes in verbs: remember, understand, apply, analyze, evaluate, and create, as shown in Figure 1. Specifically, readers should be well-guided to remember and understand words and concepts related to the materials in the pre-reading stage and then be directed to parse and analyze the ideas and their connections. Finally, it is expected that readers may reach the upper part of the “analyze” zone and even touch the objective of “evaluate”.

### 2.3. Computer technology in reading

Intelligent tutoring systems (ITS) have been increasingly used in literary education during the past two decades. Traditionally, a reading process consists of pre-reading, during reading, and post-reading activities, and intelligent reading tutoring systems built on this principle are often organized reading units that contain pre-assessment, warmup activities for comprehension guides, comprehension practice, and multiple-choice questions for post-assessment (Jones et al., 2004) or enhance the interactions during a reading with cooperative dialogs in natural languages (Shi et al., 2018; Afzal et al., 2019a,b). However, the lessons in these reading ITS are fixed, which limits their applications in the reading comprehension courses that must meet the diverse requirements of readers at different proficiency levels.

Vocabulary is a prerequisite for fruitful reading comprehension, and various computerized tools are developed to aid readers in memorizing new words. Short message services (Alemi and Lari, 2012) and mobile applications (Klimova and Zamborova, 2020) were used to build vocabulary. Empirical studies demonstrated that reading tutors improved the reading comprehension of children (Mostow et al., 2003) and their vocabulary knowledge of words (Baker et al., 2021). In addition, concepts, similar to vocabulary, are important factors that affect the outcomes of reading comprehension, and they can be learned or retained by connecting the new concept with learned concepts to form concept maps. TOM, an intelligent tutor, is developed to mine the concepts from the text and build reference concept maps automatically or semiautomatically (Boguski et al., 2019).

Based on connectivism, the learning theory that emphasizes the knowledge gained through networking and connections, peer tutoring was proposed as an effective way to enhance reading comprehension abilities (Van Keer, 2004; Blanch et al., 2012). Relying on the proposed effectiveness of peer tutoring, ITS systems based on Web 2.0 that integrated the interactions of readers were also developed to cultivate literacy of reading (Mendenhall and Johnson, 2010).

In the era of big data and artificial intelligence, new challenges arise. During the coronavirus disease 2019 (COVID-19) pandemic, many online courses were opened, but direct communications between lecturers and students were somehow blocked by the distance, as the lecturers could not view the facial expressions of the students. It is much harder for lecturers to instruct the students to proceed with their reading journey. In addition, the textbook and course materials are prepared uniformly in advance, which is unsuitable for the personalized development of reading literacy. Thus, an intelligent reading bot that alleviates the burden of instructors and automatically generates guidance for the readers is especially valuable.

## 3. Reading-related technologies in the age of deep learning

### 3.1. Pre-trained language models and performance-boosting techniques

Large pre-trained language models (LPLMs) are currently the foundational element in the applications of natural language processing. LPLMs are trained on massive language data with unsupervised or semi-supervised methods, that is, by replacing randomly selected words in a sentence with [MASK] tokens and requesting the model to predict the masked words (masked work prediction) or requesting the model to predict the next sentence (next sentence prediction). The models are trained iteratively until the preset training epochs or training objectives are reached. This training leads LPLMs to discover and represent much of the structure of human languages, assembling a broad general knowledge of the language and the world (Manning, 2022). For example, the widely used Bidirectional Encoder Representations from Transformers (BERT), released in 2019, was trained on BooksCorpus (800M words) and English Wikipedia (2,500M words) (Devlin et al., 2019); the GPT-3 (Generative Pre-training) was trained on Common Crawl, WebText2, Books1, Book2, and Wikipedia with a total of 499 billion tokens (Brown et al., 2020).

However, LPLMs are not well-suited to perform specific natural language processing tasks. They are usually fine-tuned by providing a set of examples labeled in the desired way to gain better performance (Manning, 2022), such as few-shot or zero-shot learning, including the technologies for guided reading listed below. For instance, the Stanford Question Answering Dataset (SQuAD) is a reading comprehension dataset consisting of 100,000+ questions posed by crowdworkers on a set of Wikipedia articles, where the answer to each question is a segment of text from the corresponding reading passage (Rajpurkar et al., 2016). After fine-tuning this dataset, the performance of the deep learning model on this dataset was 89.6% in the exact match (which measures whether the predicted answer is identical to the correct answer), 2.8% higher than human performance (Zhang et al., 2021). In few-shot learning, researchers only prepare a few examples for the LPLMs and then give the prompts for the model to make predictions. For example, using prompts of the form “Poor English Input: < sentence>/n Good English Output: < sentence>”, researchers give GPT-3 one human-generated correction and then ask it to correct five more (Brown et al., 2020).

### 3.2. Word sense disambiguation (WSD), named entity recognition (NER), and knowledge graph (KG)

Vocabulary building is a prerequisite for reading comprehension, as the understanding of words constitutes the foundation for understanding sentences. To accurately determine the specific “sense” of a word in a particular context, word sense disambiguation (WSD) is applied. WSD is essentially the task of determining the word sense with respect to a finite and discrete set of senses from a dictionary, a lexical knowledge base, or an ontology. It is widely used in machine translation, information retrieval, and lexicography (Agirre and Edmonds, 2007, p. 1–2). Studies showed that word sense disambiguation could facilitate second-language vocabulary learning (Kulkarni et al., 2008). WSD is also used in e-learning to improve information retrieval in the question-answering system (Hung et al., 2005) or intelligent reading through contextualized word lookup (Govindu et al., 2021).

Proper names are a special category of words called named entities in computational linguistics. Named entity recognition (NER) is the task of assigning words or phrases with tags like PERSON, LOCATION, or ORGANIZATION. They are particularly important for machine translation, information retrieval, information extraction, and question-answering systems (Indurkhya and Damerau, 2010). However, there are few studies on the efficacy of named entity recognition in learning concepts.

**Figure 2:**
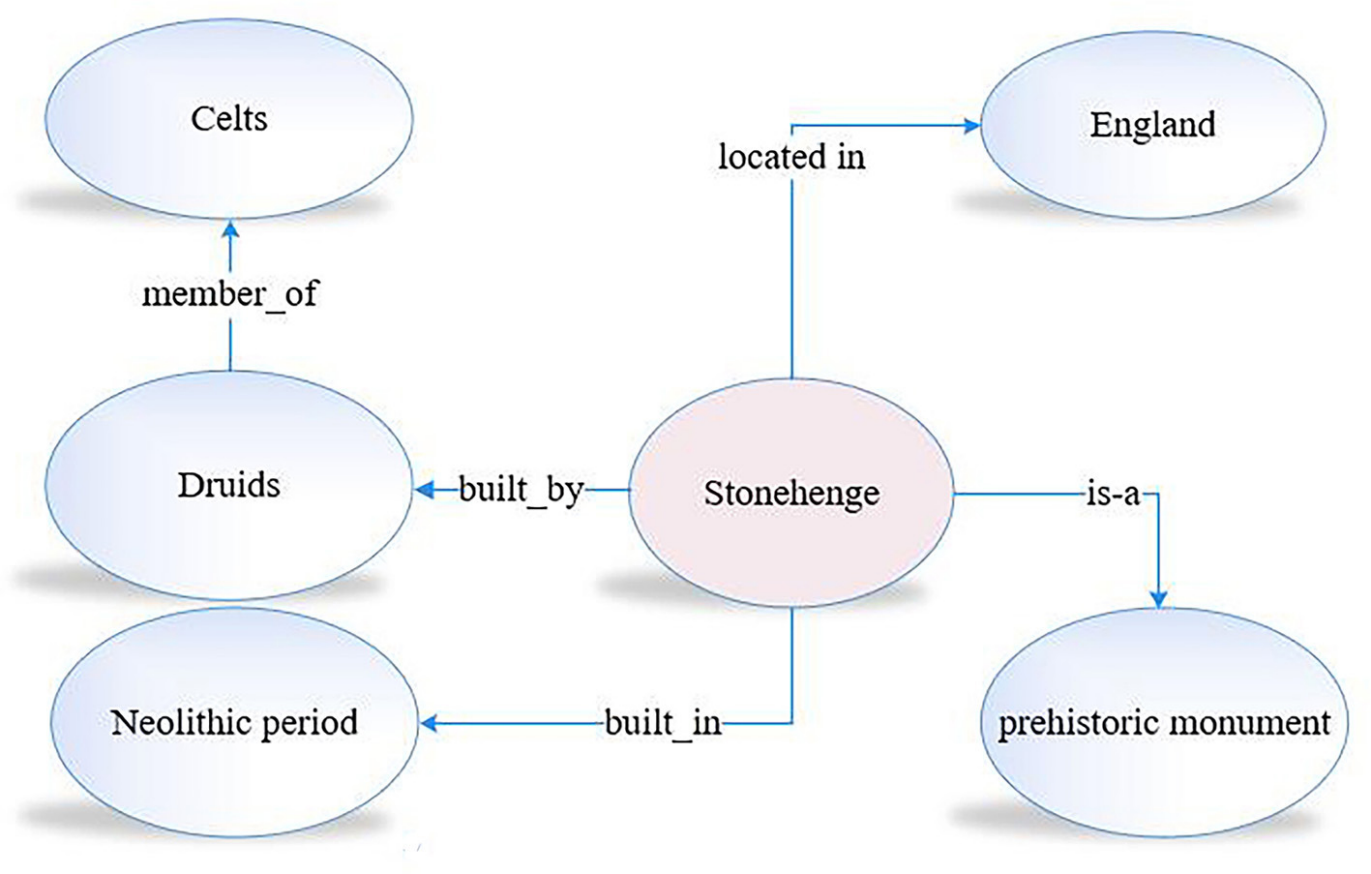
Knowledge graph fragment.

In reading comprehension, the knowledge we possess is organized into an intricate and internally consistent working model of the world based on the category system that is essential for our understanding of the world (Smith, 2004, p. 112). This type of knowledge organization is modeled as a knowledge graph, where concepts are nodes and the relationships among concepts are the edges in the graph. For example, the relationship between Stonehenge, a unique prehistoric monument, and the related concepts, such as Druids, England, and the Neolithic period, are modeled in a knowledge graph, as shown in Figure 2, which offers an excellent way for inquisitive exploration of numerous related concepts. In the knowledge graph, we express our knowledge of Stonehenge by representing the entities as nodes in the graph and expressing relationships between entities *via* edges that connect these nodes.

**Figure 3:**
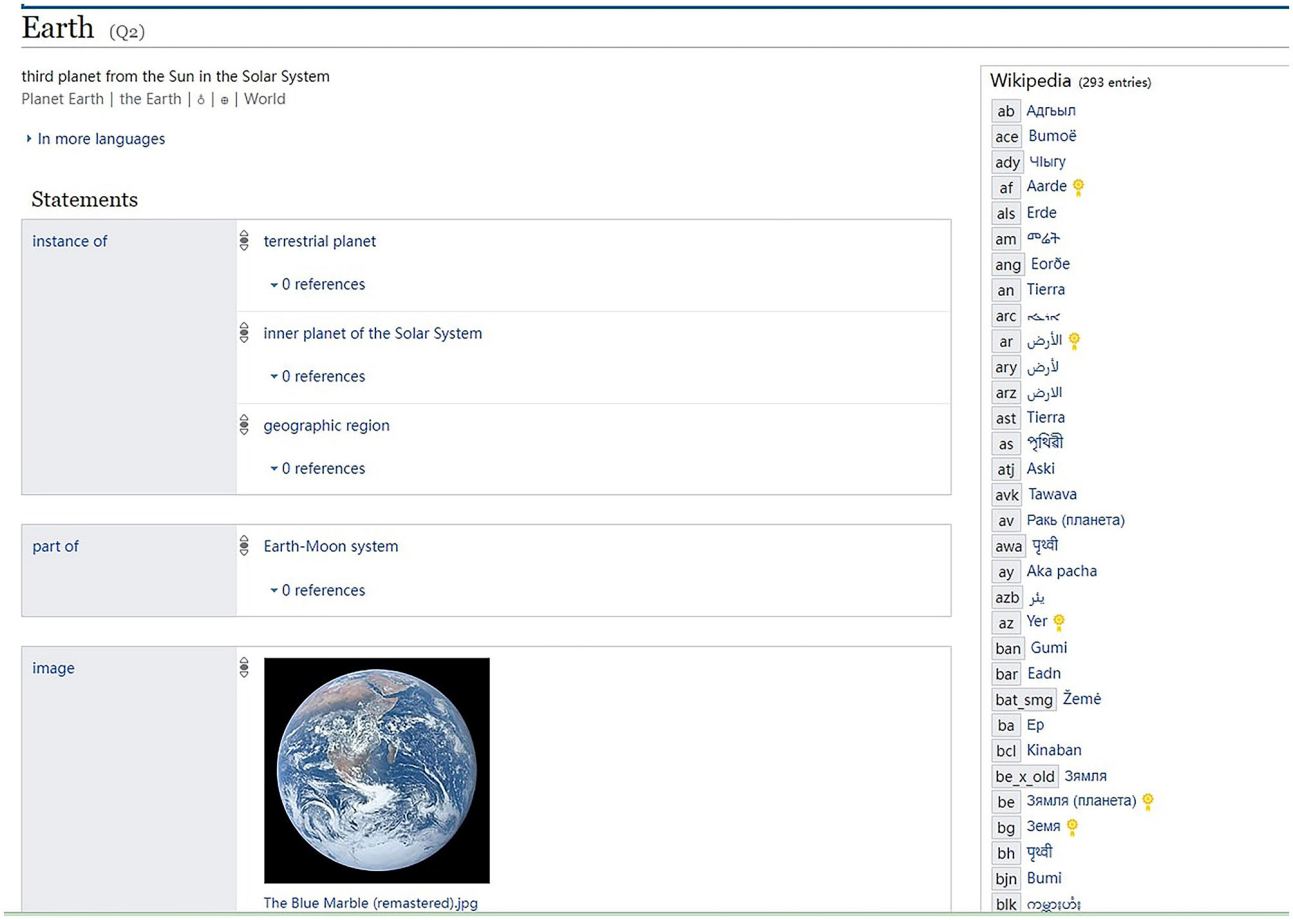
Earth in Wikidata (https://www.wikidata.org/wiki/Q2).

As knowledge graphs constitute an ideal platform to organize knowledge, huge knowledge graphs are built by crowd-sourced workers. Wikidata, a companion to Wikipedia that provides linked data for Wikipedia documents, is a well-known example of the knowledge graph, which turns the unstructured text in Wikipedia into structured knowledge, as shown in Figure 3. In Wikidata, images and videos concerning the Earth are all connected to this central concept, together with its relationship with other concepts, such as the “part of” relation with the Earth-Moon system.

Knowledge graphs are important knowledge repositories for educators and learners to understand concepts and their relationships. Studies showed that the presentation of concepts and their relationships helps learners with vocabulary building (Sun et al., 2020) or making learning plans for the computer science major (Li et al., 2019).

### 3.3. Text simplification

Long and complex sentences often pose difficulties in reading comprehension, especially for readers with low reading fluency. Text simplification, in natural language processing, aims at producing a simplified version of the original sentence to facilitate reading and understanding. Studies showed that the simplified text could benefit foreign language learners (Yano et al., 1994), leading to better text comprehension, particularly for people at lower English proficiency levels (Rets and Rogaten, 2021) and children with low reading fluency and weak cognitive skills (Javourey-Drevet et al., 2022).

Text simplification was conducted by automatically adapting texts into shorter contents of simpler linguistic structures with simplification rules (Watanabe et al., 2009). With recent advances in deep learning, better text simplification models were developed to break down a complex source sentence into a set of minimal propositions with a clearly labeled discourse tree to preserve the coherence structure based on rhetorical structure theory (RST) (Niklaus et al., 2019). Another strategy is to simplify the sentences based on controllable text generation, in consideration of the attributes of the output sentence such as its length, the amount of paraphrasing, lexical complexity, and syntactic complexity (Martin et al., 2020). For example, the sentence “He settled in London, devoting himself chiefly to practical teaching.” should be simplified to a shorter sentence “He teaches in London” (Martin et al., 2020). Text simplification may be used to help readers with reading difficulties, as demonstrated by a sentence simplification tool for children with low reading skills (Barlacchi and Tonelli, 2013).

### 3.4. Question generation (QG)

As questions are standard constituents in testing reading comprehension, question generation becomes an indispensable part of guided reading. Question generation (QG) is used to automatically generate questions for given sentences, paragraphs, or documents, which has wide applications for assessments and self-assisted learning (Kurdi et al., 2020), avoiding the necessity of manual work by teachers. With the tremendous potential of reading, question generation has been an active research field in natural language processing. In 2010, the first challenge on question generation was held to evaluate the performance of models in generating questions from sentences or paragraphs. Based on the lexical, syntactic, and/or semantic information, Aldabe et al. (2006), Das et al. (2016), Huang and He (2016), and Gilbert and Keet (2018) proposed a rule-based or template-based question generation system to generate questions.

The advent of deep-learning technologies boosted the performance of question generation. A much greater effort was placed into generating diverse and hard questions. High-quality questions were generated based on the pre-trained language models (Wang et al., 2018; Kumar et al., 2019; Pan et al., 2020; Cheng et al., 2021) and, in particular, for educational purposes (Stasaski et al., 2021; Rathod et al., 2022; Zou et al., 2022). In addition, for multiple-choice questions, distractor generation also received due attention (Liu et al., 2005; Susanti et al., 2018; Gao et al., 2019; Qiu et al., 2021; Ren and Zhu, 2021; Zhang and VanLehn, 2021). Despite the promising results of these question generation models, the applications based on these models were not adequately addressed because the performance of most models was measured on specified datasets, and their implementation required considerable effort and knowledge in computing.

### 3.5. Automatic short answer grading and automatic essay scoring

Automatic short answer grading (ASAG) or automatic short answer assessment is the task for the automatic scoring of a particular answer to a short question. As human grading of open-ended questions is time-consuming and labor-intensive, research on automatic short answer/essay assessment has been active since 1966 (Page, 1966). C-rater was developed by ETS Technologies to score short answers and measure the understanding of content materials, with the correct answer created by a content expert (Leacock and Chodorow, 2003). Similarly, an E-rater was also developed to score essays, which was evaluated on Test of English as a Foreign Language (TOEFL) exams and recommended for operational use (Ramineni et al., 2012).

After the emergence of large pre-trained language models, BERT, GPT, and their variants were evaluated to boost the performance of automatic short answer grading (Gaddipati et al., 2020; Condor et al., 2021; Chang et al., 2022). In addition, with human involvement, the study showed that the automatic short answer assessment can achieve accuracy equivalent to that of teaching assistants (Schneider et al., 2022). Automatic essay scoring (AES) also received intensive studies, with performance boosted by sentence BERT (Chang et al., 2021) and the joint learning of multi-scale essay representation (Wang et al., 2022b).

## 4. Model mapping and implementation for reading bots

### 4.1. A theoretical model applicable in reading assisted with deep-learning technology

Reading models from different scholars reveal the science of reading from distinct perspectives, emphasizing the integration of various skills. For example, Scarborough's reading rope explicitly stated that reading comprehension utilizes a combination of skills, including word recognition and language comprehension, which cover background knowledge, vocabulary, language structures, verbal reasoning, and literacy knowledge. With various reading activities, these skills are trained and polished in the reading process, which can be improved with deep-learning technologies.

**Figure 4:**
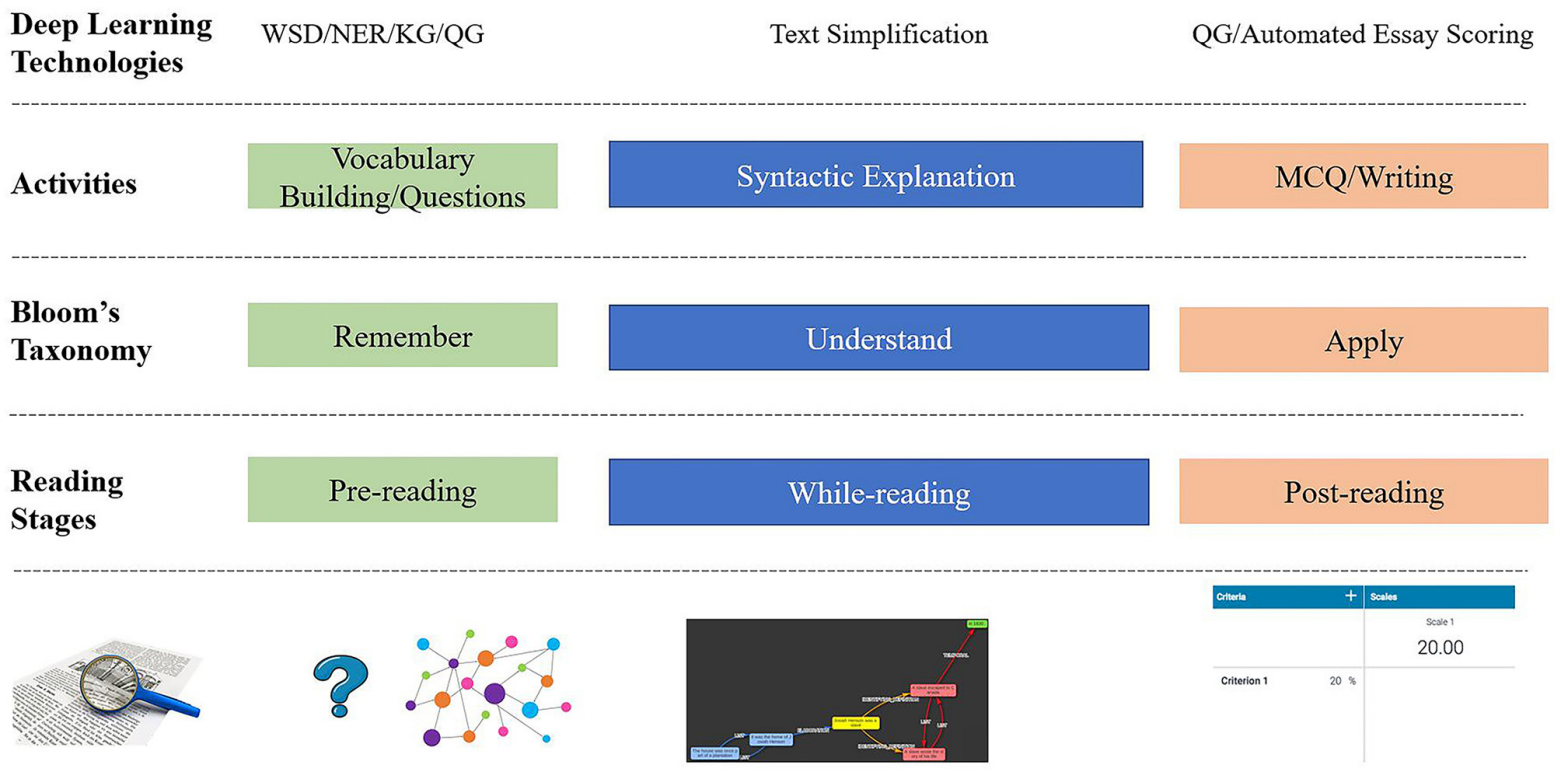
Mappings between reading stages and deep-learning technologies.

The activities in reading comprehension are divided into the following three stages: pre-reading, while-reading, and post-reading. We created a mapping between the common activities in the reading processes and related deep-learning technologies using Bloom's taxonomy of learning objectives, as illustrated in Figure 4.

Deep-learning technologies and concepts in the knowledge graph expose hidden structures of the unstructured text and background schemata from pre- to post-reading. Finally, computerized essay scoring evaluates text summaries of readers.

The purpose of pre-reading activities is to prepare the readers for the reading materials, and they are often named warm-up activities. Warm-up activities include vocabulary learning in which pictures or concept maps are used to assist readers and guide them in building a schema. For vocabulary learning, instructors often prepare the new word list and related background knowledge, which are learned first by the readers before they read the text materials. With the reading bots, automatic vocabulary filtering based on word difficulty levels can be applied to obtain new vocabulary. In addition, frequent multiword expressions, in particular, the named entities such as locations or company names, are extracted with named entity recognition. These extracted words or multiword expressions are connected with external knowledge in the pre-built knowledge graphs. From the knowledge graph, readers can obtain related images and audio files and form an expanded scenario, thus triggering the schemata for the forthcoming text materials. Another warm-up activity in pre-reading is the guiding questions. Guiding questions are generated using a two-step procedure. In the first step, automatic summarization of the reading materials is conducted to shorten the text to a reasonable size. Then, question generation is performed on that summary to obtain questions.

While-reading is a crucial stage in reading comprehension in which readers absorb the knowledge from the reading materials and integrate it with the existing knowledge in their minds. As stated in Bloom's taxonomy, it is the primary stage that elevates the readers from remembering to understanding the reading materials. Significant challenges in this stage include difficulties in analyzing the structure of long and complex sentences. To manage this challenge, we propose using text simplification to assist readers in untangling complex sentences.

Post-reading is the stage where readers review the reading materials and check their understanding to elevate the learning to the higher levels in Bloom's taxonomy, such as applying, analyzing, and evaluating. A quiz automatically generated with multiple-choice questions is arranged to assist the readers in checking their understanding. The readers may be requested to write a short essay concerning the reading materials. To manage these tasks automatically, we propose to (1) arrange fill-in-the-blank questions to check the memory of readers of the reading materials; (2) prompt the readers with multiple-choice questions to validate their understanding; and (3) use the automated essay scoring engine to help readers evaluate their writing independently.

### 4.2. Design and implementation of reading bots

**Figure 5:**
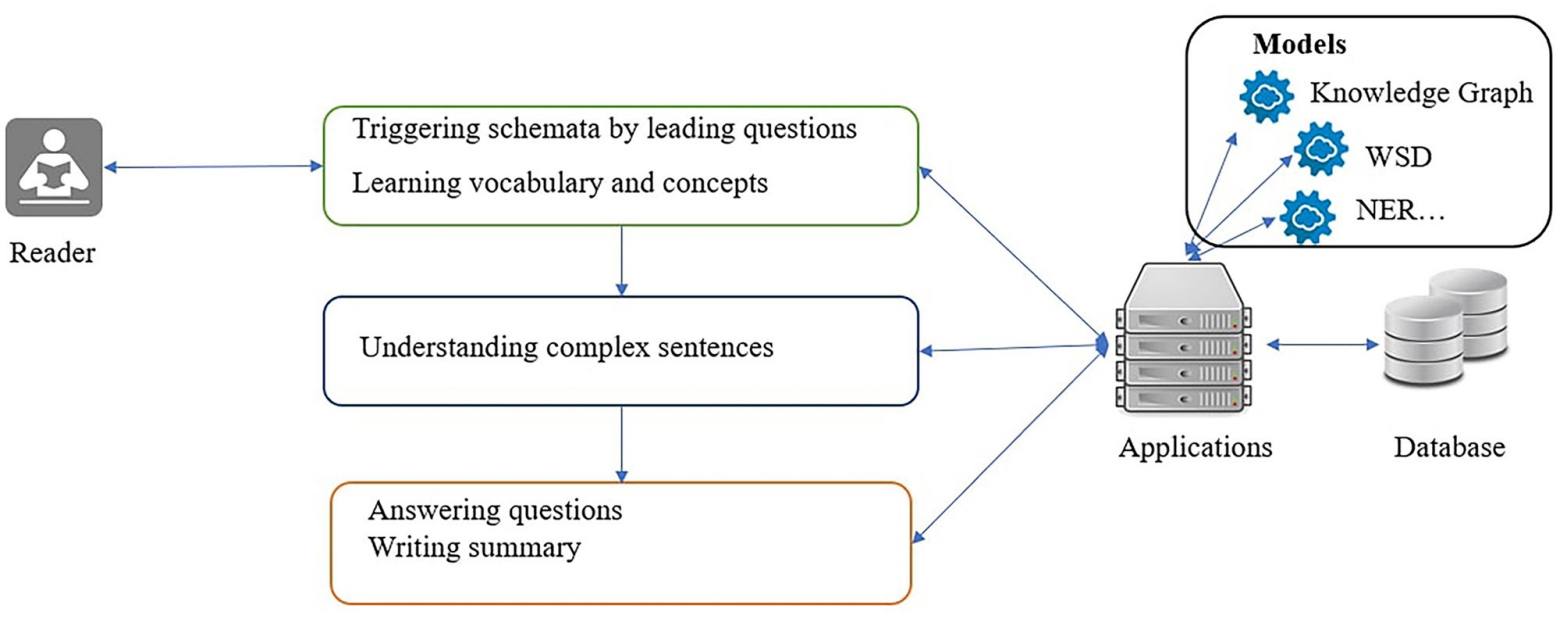
Interactions between readers and the reading bot.

We created a reading bot system to validate the applicability of the current deep-learning technologies in guided reading. The reading bot system uses the existing open-source deep learning models for word sense disambiguation, question generation, text simplification, and automated essay scoring. For the knowledge graph, the system uses the external service provided by BabelNet (Navigli and Ponzetto, 2012). BabelNet 5.1 is an innovative multilingual encyclopedic dictionary which connects concepts and named entities in a very large network of semantic relations and is made up of ~22 million entries obtained from the automatic integration of WordNet (Miller, 1995), Wikipedia, ImageNet (Fei-Fei et al., 2010), VerbAtlas (Di Fabio et al., 2019), and other existing knowledge graphs and lexicons (Babelscape, 2022). The overall interactions between the readers and the reading bots are shown in Figure 5.

The readers are presented with web pages to guide them from the pre-reading stage to the post-reading stage. Reader interactions with the reading bots are recorded and saved in the databases for learning analytics.

The system consists of four layers, namely, front-end webpages, where readers access the functionalities of the system; the application logic layer, which redirects the requests from readers to the appropriate resources and saves the interactions into a database; the restful resources layer, which exposes the functions of deep learning models as RESTful services; and external resources, which are the knowledge bases provided by other websites *via* application programming interfaces (API).

**Figure 6:**
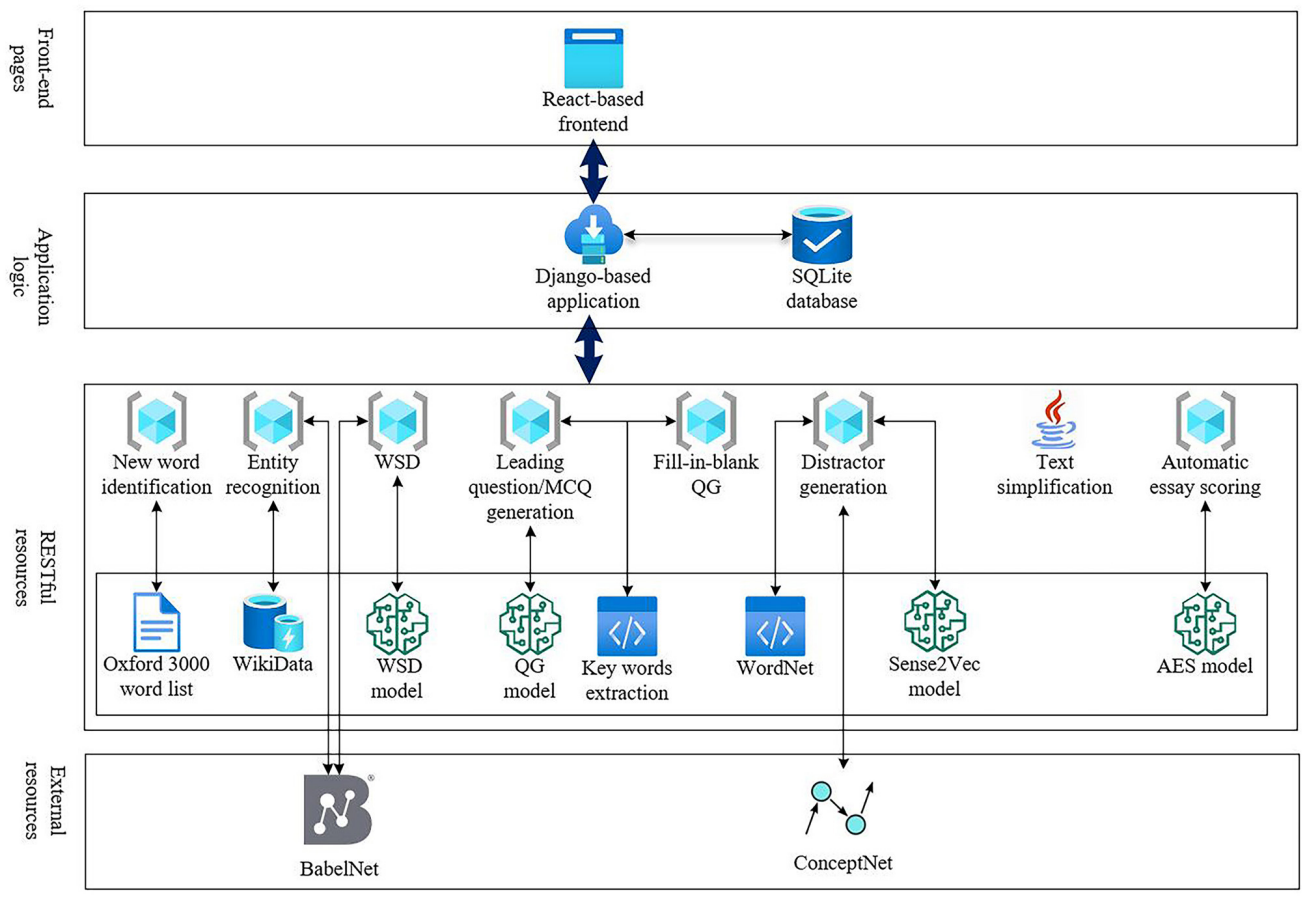
Architecture of the reading bot.

The reading bot system is a web application designed based on the principle of separation of concerns, which divides the functions into separate sections. The front-end web pages are developed based on React, an open-source JavaScript library for building user interfaces sponsored by Facebook. The backend is based on Django,^2^ a Python web framework that offers plenty of out-of-box functionalities, including database access, user authentication, and group management, and the interactions with readers are stored in an SQLite^3^ database. Advanced features provided by deep learning models are exposed as RESTful services (Fielding, 2000), with the support of FastAPI.^4^ These models are served independently as separate services, and the features can be easily enhanced with the recent models if necessary. Apart from the Java-based text-simplification service, other services are pure Python. External resources, including BabelNet and ConceptNet, are incorporated into the system with their application programming interfaces (API). The whole system is supported by microservices instead of being a monolith, as shown in Figure 6.

When using the reading bot system, the words in the reading materials are first filtered *via* a word list based on the language proficiency level of the readers, such as B1, B2, or C1 in the Common European Framework of Reference for Languages (CEFR) (Council of Europe, 2001). Currently, the word list is based on the Oxford 3000 word list (Oxford University Press, 2019). Then, readers are prompted with guiding questions generated with a question generation (QG) model trained on the SQuAD dataset, with the answer extracted by the machine learning algorithm from pke, an open-source Python-based keyphrase extraction toolkit (Boudin, 2016). In addition, the specific meaning of the new words in the context is identified after word sense disambiguation with the BERT-based WSD (word sense disambiguation) model (Yap et al., 2020) and WordNet (Miller, 1995). The named entities are recognized with spaCy (Honnibal and Montani, 2017), and a named entity linker is used to look up these entities from the Wikidata database. After extracting the new words and entities, the system retrieves the Uniform Resource Locators (URL) of the related images and audio for both the new words and the entities *via* the API of BabelNet. In the while-reading stage, readers can simplify the complex sentences and view the simplification results with DISSIM, a Discourse-Aware Syntactic Text Simplification Framework for English and German (Niklaus et al., 2019), which breaks the complex sentence into simpler ones and explicitly labels the discourse relations. Finally, readers are challenged with fill-in-the-blank questions, multiple-choice questions, and writing tasks in the post-reading stage. Distractors in the multiple-choice questions are generated based on the semantic relationships in the WordNet or ConceptNet, resorting to the Sense2Vec model (Trask et al., 2015) for similar words in the vector space if no candidate distractors can be found from the two; the composition written by readers will be auto-graded by the BERT-based automatic scoring engine that was trained on automated essay scoring (AES) dataset (Wang et al., 2022b). AES is a set of high school student essays along with scores generated by human expert graders (Hewlett Foundation, 2016).

**Figure 7:**
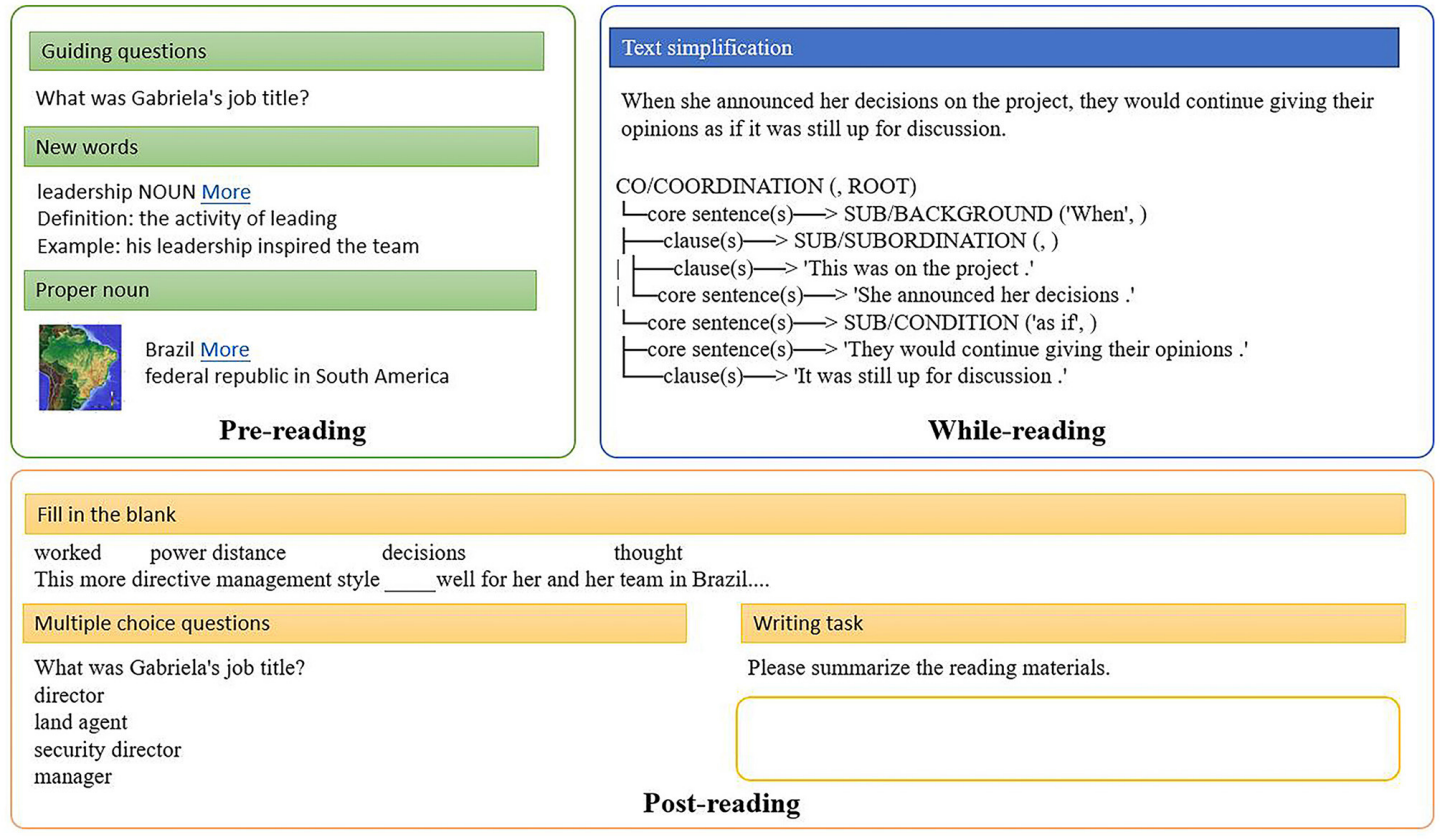
Sample results in the pre-reading, while-reading, and post-reading stages.

As illustrated in Figure 7, in the pre-reading stage, guiding questions are generated based on the summarization of the reading materials. Words in the vocabulary and proper nouns are obtained after filtering the reading materials with the Oxford 3000 list and identifying the entities. Images are retrieved from BabelNet, and readers can click “More” to visit the entry on the website of BabelNet. In the while-reading stage, complex sentences can be simplified with the text simplification model. For example, “*When she announced her decisions on the project, they would continue giving their opinions as if it was still up for discussion*.” is broken into core sentences (“*She announced her decisions*”, “*They would continue giving their opinions*”) and clauses (“*This was on the project*”, “*It was still up for discussion*” and labeled with discourse relations (SUB/BACKGROUND *when*, SUB/CONDITION *as if* ). In the post-reading stage, fill-in-the-blank questions and multiple-choice questions are automatically generated. In addition, readers are prompted with a text input to write their summaries of the reading materials, which the automatic essay scoring engine will rate.

## 5. Case studies

Deep learning models, despite their reported performance on the selected datasets, have not been widely tested yet. To understand their performance in real-world scenarios, we conducted experiments with the openly accessible articles from Learning English on the website of the British Council.^5^ We selected 8 articles from the A2 reading, 10 articles out of 12 from the B1 reading, and 10 articles out of 12 from the B2 reading on the website, excluding incomplete articles, articles in tables, or articles in colloquial language. The complete list of the articles in the evaluation is presented in Table 1.

**Table 1:** Articles in the case studies.

**Title**	**Level**
A message to a new friend	A2 reading
An email from a friend	A2 reading
An end-of-term report	A2 reading
An invitation to a job interview	A2 reading
Choosing a conference venue	A2 reading
English course prospectus	A2 reading
Professional profile summaries	A2 reading
Study skills tips	A2 reading
A flyer for a gym	B1 reading
A travel guide	B1 reading
An email request for help	B1 reading
Digital habits across generations	B1 reading
Encyclopedia entry	B1 reading
How to spot fake news	B1 reading
Innovation in business	B1 reading
Robot teachers	B1 reading
Social media influencers	B1 reading
The legend of fairies	B1 reading
A short story extract	B2 reading
An email from a friend	B2 reading
Asteroids	B2 reading
Cultural expectations and leadership	B2 reading
Millennials in the workplace	B2 reading
Star Wars and the hero myth	B2 reading
The buy nothing movement	B2 reading
The sharing economy	B2 reading
Why bridges collapse	B2 reading
Work–life balance	B2 reading

With the 28 articles selected, we evaluated three features of this system: word sense disambiguation, named entity identification, and question generation. As vocabulary learning is an important prerequisite for reading and vocabulary instruction improves reading comprehension (Castles et al., 2018), both word sense disambiguation and named entity identification were evaluated to explore their strengths and weaknesses and check their applicability in real-world applications, in consideration of the reported performance of the word sense disambiguation model that was nearly 80% in F1 scores (a measure of the accuracy of a model on a dataset). Question generation is a relatively hot subject in natural language processing due to its value in education, chatbots, and other fields. Considering the good human evaluation results reported on the specific datasets and their potential in guided reading, we evaluated the performance of the question generation model in the system, exploring the practicality of the models in real-world applications and detailing their limitations.

In the evaluation, the new words were extracted from the text with a rule-based algorithm against the words at the proficiency level immediately below the reading level. For example, for the articles extracted from the B2 reading section on the British Council, the proficiency level B1 was used for filtering the new words.

The summary of the reading materials and the results are presented in Table 2. The question generation model in the system generates three or more leading questions for each article. However, the number of leading questions and the number of multiple-choice questions (MCQ) do not match, indicating that the generation of distractors in the system needs to be improved because the system drops a question if there are not enough distractors for that question.

**Table 2:** Summary of the reading materials and the model results.

**Level**	**A2**	**B1**	**B2**
No. of articles	8	10	10
Avg. article length (in words)	194	356	470
Total new words	105	167	104
New words with correct senses	85	145	97
Ratio of words with correct senses	80.95%	86.83%	93.27%
Total entities	52	53	57
Entities with correct senses	33	33	38
Ratio of entities with correct senses	63.46%	62.26%	66.67%
No. of leading questions	31	37	38
No. of MCQ	23	33	33

We manually checked the correctness of the sense of the identified new words and entities. As shown in Table 2, the ratios of the words with correct senses in A2, B1, and B2 are above 80%, being 80.95%, 86.83%, and 93.27%, respectively. The ratios of the entities with correct senses in A2, B1, and B2, however, are below 70%, being 63.46%, 62.26%, and 66.67%, respectively. Table 3 presents samples of good and bad cases. It is clear that “collapse” and “Airbnb” are correctly identified and associated with their corresponding meanings. However, “mission,” a polysemic word, is incorrectly associated with the meaning related to missionaries. Similarly, “Lot” in “Lots of love” is incorrectly traced to the person Lot in the Book of Genesis due to the capitalized “L.”

**Table 3:** Good and bad cases for new word and entity identification.

**Item**	**Good case**	**Bad case**
Word	Collapse	Mission
Context	*Luckily, this kind of collapse is relatively infrequent*	*From there I was on another three-month mission to oversee..*.
Meaning	A natural event caused by something suddenly falling down or caving in	An organization of missionaries in a foreign land sent to carry on religious work
Entity	Airbnb	Lot
Context	*Companies like Airbnb act as a middleman for…*	*Lots of love*
Meaning	Online platform for rental accommodations	Person mentioned in the biblical Book of Genesis and the Quran

**Figure 8:**
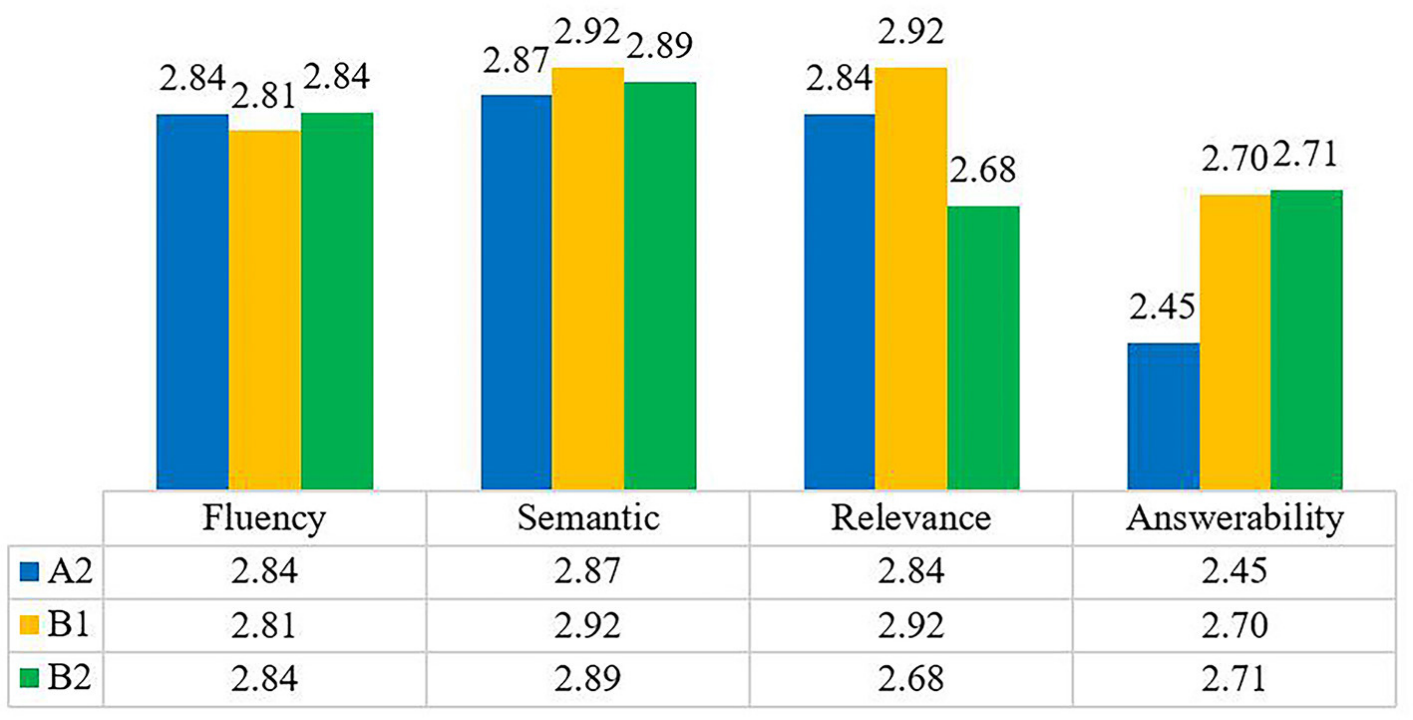
Question generation evaluation results.

We also evaluated the quality of the generated leading questions and multiple-choice questions with a three-point rating (bad, fair, good) in the metrics of fluency, semantics, relevance, and answerability as described in Table 4. According to the evaluation results in Figure 8, the scores on fluency, semantics, relevance, and answerability are above 2.60, indicating that most of the questions generated are grammatically correct, semantically clear, and related to the passage. However, the score on answerability for A2 is 2.45, indicating that more efforts should be made in this respect in the future.

**Table 4:** Human evaluation metrics with description (Zou et al., 2022).

**Criteria**	**Rating**	**Score**	**Description**
Fluency (grammatical correctness)	Bad	1	Not readable due to grammatical errors
	Fair	2	Contain few grammatical errors but not affect the readability too much
	Good	3	Free from grammatical errors
Semantic (clarity and logical correctness)	Bad	1	Have obvious logical/common-sense problem or indecipherable
	Fair	2	Have some semantic ambiguities
	Good	3	Semantically clear
Relevance (to the passage)	Bad	1	Totally irrelevant
	Fair	2	Part of the question is irrelevant
	Good	3	Relevant
Answerability	Bad	1	Not answerable
	Fair	2	Not sure about the correct answer
	Good	3	Can be answered by the right answer

Table 5 presents a sample of good and bad questions. In the article *Star Wars and the hero myth*, the leading question “*What is the film's structure called?*” sounds like an ordinary question, and its answer is contained in the text “*both films follow a structure that pre-dates all Hollywood films, that of the 'hero myth'*” in the article. “*Experts predict robots will transfer what?*” sounds somewhat awkward, and “*What makes a good what?*” may confuse readers. In addition, for “*What is strange about London?*” and “*Where is John Sutter based?*”, both London and John Sutter are missing in the articles with the same title *An email from a friend*. The reasons behind the performance difference could be that the models were trained on questions from Wikipedia articles, so they tended to perform better on similar articles.

**Table 5:** Sample of good and bad questions.

**Type**	**Question text**	**Article title**	**Level**
Good question	What is the film's structure called?	Star Wars and the hero myth	B2
Question with low fluency score	Experts predict robots will transfer what?	Robot teachers	B1
Question with low semantic score	What makes a good what?	Study skills tips	A2
Question with low relevance score	What is strange about London?	An email from a friend	A2
Question with low answerability score	Where is John Sutter based?	An email from a friend	B2

## 6. Challenges and limitations

One prominent issue concerning our proposal is the performance of the models. It was reported that the performance of the word sense disambiguation model used in our study achieved 79.5% in the F1 score over the five-word sense disambiguation datasets (Yap et al., 2020). Its performance on open-domain materials, as indicated in our evaluation, was below 90% accurate. The performance of named entity recognition was even worse, being < 70% accurate in our evaluation. DISSIM, the text simplification model used in our study, claimed to have a precision of ~90% and reach an average precision of ~70% for the classification of the rhetorical relations between them (Niklaus et al., 2019), which was not good enough for practical deployment. The performance of the automatic essay scoring engine was also below 90% in quadratic weighted kappa (QWK) metrics (Wang et al., 2022b). Quadratic weighted kappa (Cohen, 1968) is a common measure for evaluating an automatic essay scoring engine that measures the agreement between the scoring results of two raters. This situation is like the earlier application of machine translation. It can help educators and readers, but human intervention is required for better results.

Moreover, the question generation model was trained on SQuAD, a dataset consisting of 100,000+ questions posed by crowd workers on a set of Wikipedia articles (Rajpurkar et al., 2016). Thus, the questions generated by the model were the easiest questions that could be answered by looking up relevant parts of the text without deep thinking, which limited the test of understanding of readers at the low level of Bloom's taxonomy. In addition, our evaluation showed that there were still some errors in the questions generated in terms of fluency, semantics, fluency, and answerability, which should be handled properly before the deployment in real-world scenarios.

The second issue with the performance of the models is their domain transfer capabilities. As traditional machine learning models are trained based on the assumption that the training and testing data are identically distributed, when the probability distributions of the training data and the testing data are different, the performance of the models often deteriorates (Quiñonero-Candela et al., 2009). However, it is expensive and even prohibitively impossible to collect the data from all possible domains to train ML models (Wang et al., 2022a). Currently, the question generation models and the automatic essay scoring models are trained on specific datasets. Their performance may deteriorate considerably if they are used to process materials that differ widely from the datasets in the training. To alleviate the problems, it is necessary to fine-tune the model. For example, consider the automatic scoring model. Each line in the file for training is in the format of one composition and its score. Educators may prepare the data in the same format as the compositions and the scores rated with their own scoring rubrics, fine-tune the model, and obtain better performance. Similarly, question generation models and other models can be fine-tuned with domain-specific data. In addition, as the current system relies on existing knowledge graphs, a domain-specific knowledge graph may be built or used to offer readers insights into the relations among proper names in a particular domain. For example, in biology, the Gene Ontology (GO) knowledgebase (Ashburner et al., 2000; Carbon et al., 2021), the largest source of information on the functions of genes in the world, may be used for reading materials in the biology field.

Another challenging aspect of deep learning is its explainability, which is a crucial feature for the practical deployment of AI models (Barredo Arrieta et al., 2020). Deep learning models comprise hundreds of layers and millions of parameters, which makes deep neural networks (DNNs) considered complex black-box models (Castelvecchi, 2016). Researchers in natural language processing have already paid attention to this issue. They designed probing tasks to capture the linguistic features of sentences and the embedding generated by deep learning models (Conneau et al., 2018) or to understand how the lexical information from words in context is captured by deep learning models (Vulić et al., 2020). In addition, a unified framework for interpreting predictions called SHapley Additive exPlanations (SHAP) was developed to visualize the importance of each feature for a particular prediction (Lundberg and Lee, 2017). However, the research on explainability is in the initial stage, with the reasoning processes of the deep learning models still inside a black box that cannot meet the requirements for real-world applications. For instance, for the automatic essay scoring engine, a simple score is insufficient for readers. They want to know their shortcomings in a detailed report. For the question generation, educators may want to know why and how the question is generated and what knowledge is tested.

## 7. Conclusion

This study investigates the advances in deep-learning technologies, particularly natural language processing technologies, which are mostly related to human reading. It further explores their applications under the guidance of well-known reading models. The study uses publicly accessible models and platforms to demonstrate the potential of deep-learning technologies in guided reading, including word sense disambiguation, named entity recognition, knowledge graphs, text simplification, question generation, and automatic essay scoring. With the design and implementation of a reading bot system based on the mappings between three reading stages and the corresponding deep-learning technologies, the study not only highlights the effectiveness of such technologies but also points out their limitations based on the hands-on implementation of the related deep learning models and the evaluation of these models with 28 articles. Performance and explainability are among the important limitations that hinder the practical deployment of deep learning models. In the future, with more advances in deep learning, text-to-image generation or text-to-video generation may be used to create a live scene for readers to understand the reading materials better. Moreover, the explainable AI can also pinpoint the specific weaknesses of readers for improvement. In this way, we will not only reduce the tremendous labor required for preparing a successful reading journey but also improve the effectiveness of human reading and enhance knowledge transfer.

## Data availability statement

The original contributions presented in the study are included in the article/Supplementary material, further inquiries can be directed to the corresponding author.

## Author contributions

HZ contributed to conception. BH drafted the manuscript. JD critically revised the manuscript. All authors contributed to the article and approved the submitted version.

## Footnotes

^1^ https://www.britishcouncil.org/

^2^ https://www.djangoproject.com/

^3^ https://www.sqlite.org/index.html

^4^ https://fastapi.tiangolo.com/

^5^ https://learnenglish.britishcouncil.org
