# Supplementary material for: Reading bots: The implication of deep learning on guided reading
Source: Front Psychol. 2023 Feb 6;14:980523. doi: 10.3389/fpsyg.2023.980523 (PMC9939479; doi:10.3389/fpsyg.2023.980523)
Supplement: Supplementary file 1 [file Table_1.DOCX]

## Appendix I Description of the Reading Bots Prototype

The prototype of the reading bots consists of two features: guided reading, and reading history.

After readers input the text in the reading materials and select the proficiency level, they can click **Start Reading** to go to the **Pre-reading** page.


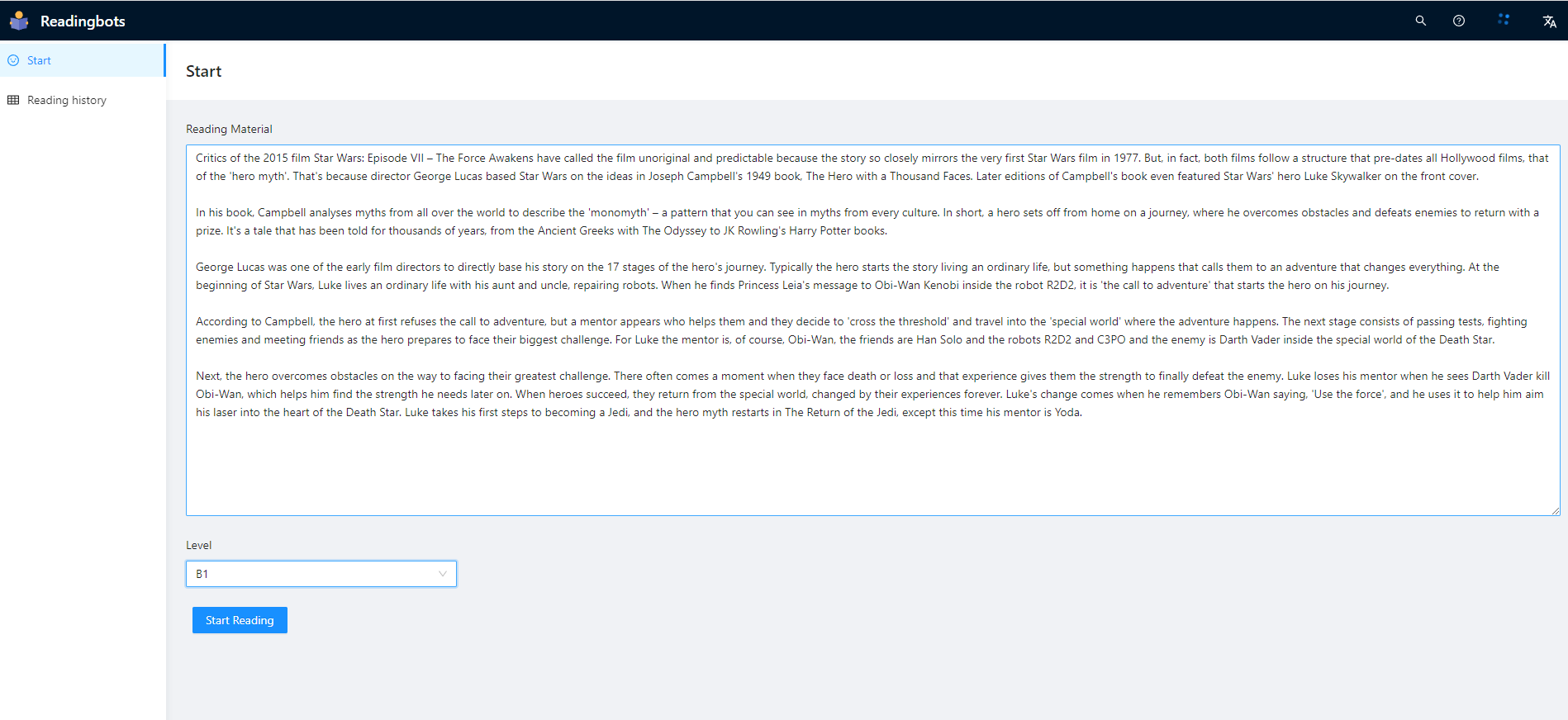


Figure 1 Start Reading Page

In the **Pre-reading** page, the guiding questions, vocabulary, and proper nouns are automatically generated or identified to prepare the necessary knowledge and context for the while-reading stage.


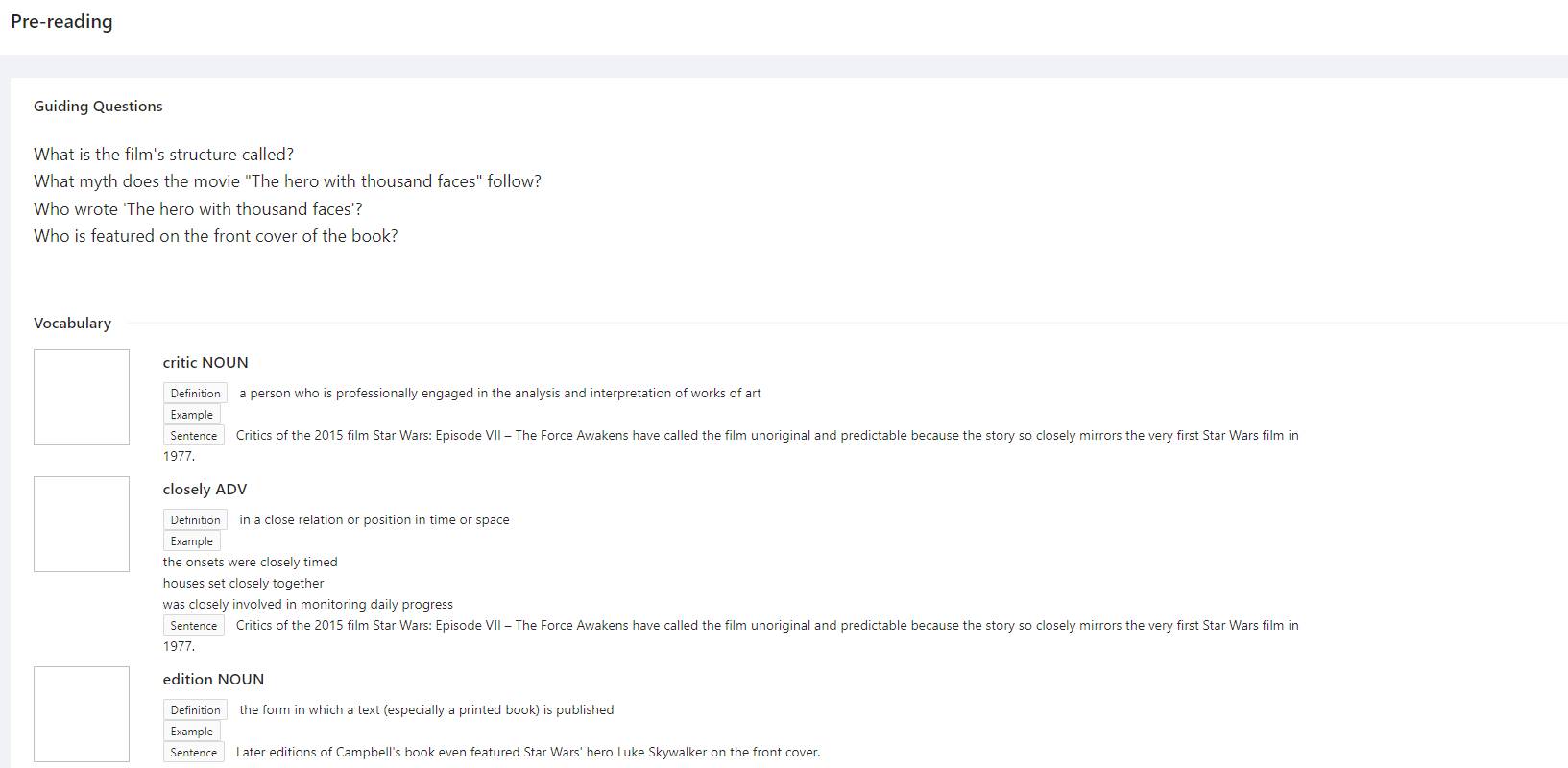


Figure 2 Pre-reading page (I)


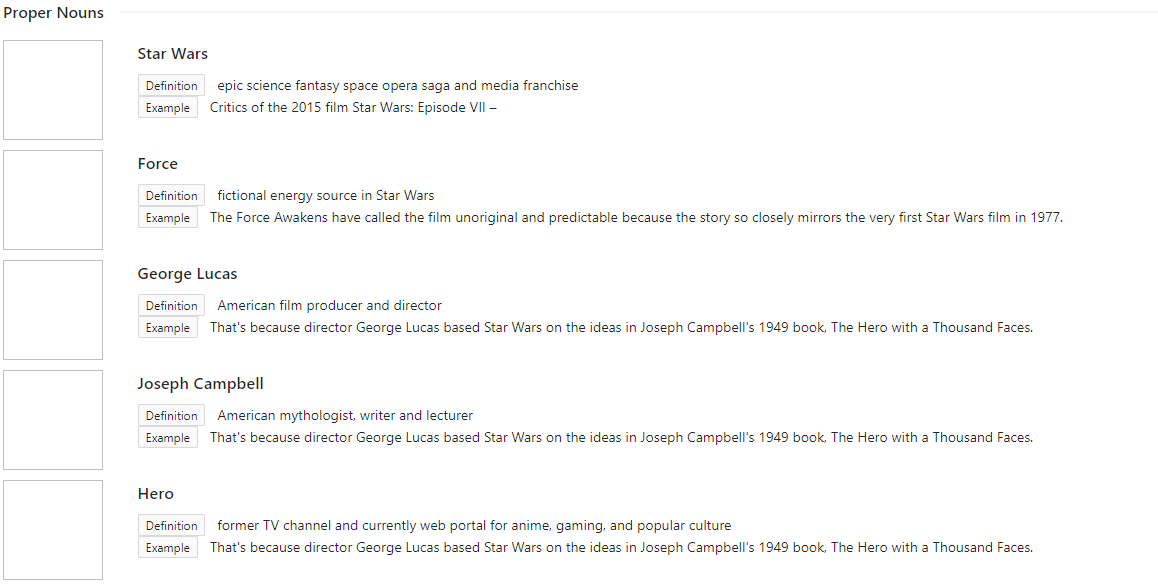


Figure 3 Pre-reading page (II)

In the **While-reading** page, after selecting a sentence and clicking **Simplify Sentence**, readers can view the simplification results.


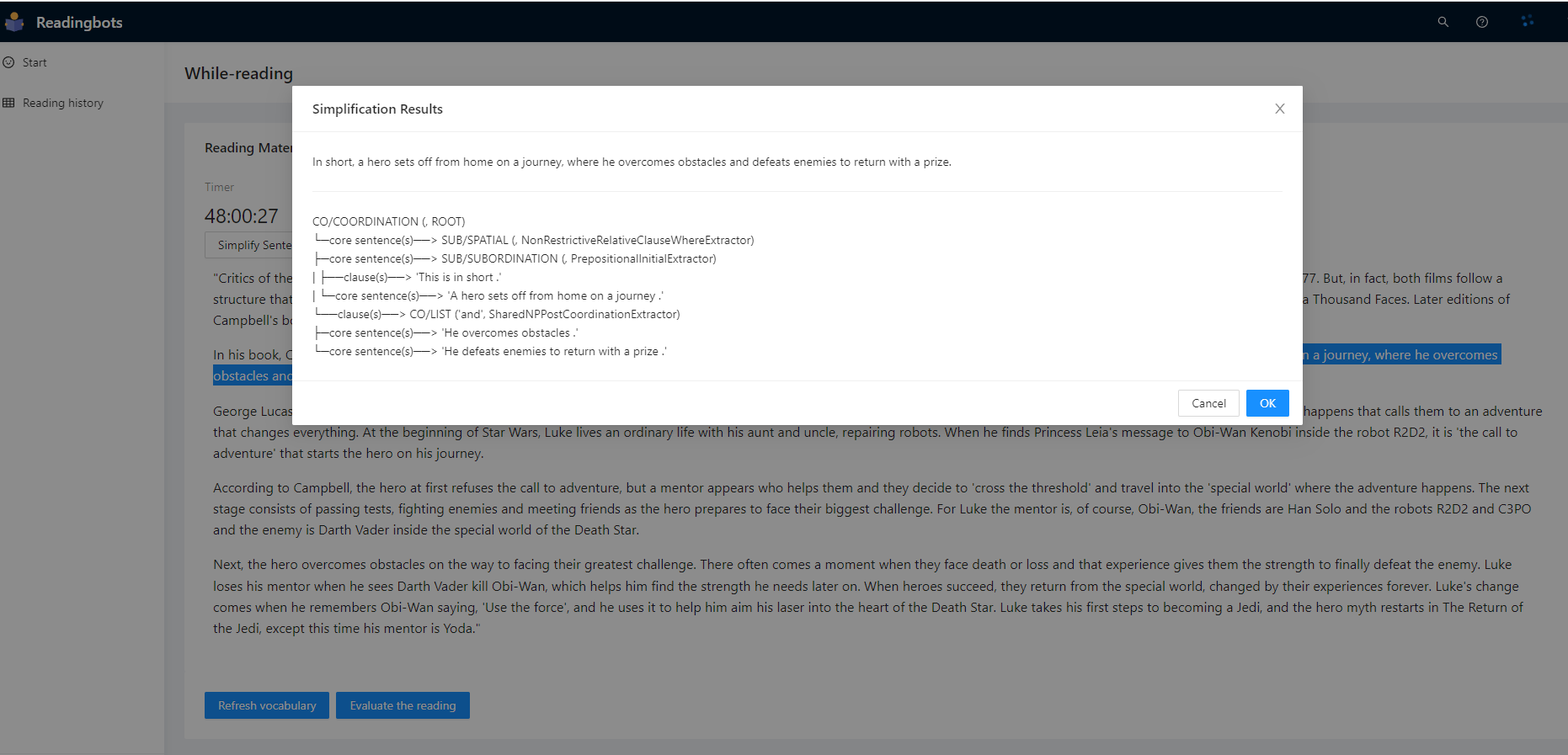


Figure 4 While-reading page

In the **Post-reading** page, readers are prompted with fill-in-the-blank questions, multiple choice questions and writing tasks.


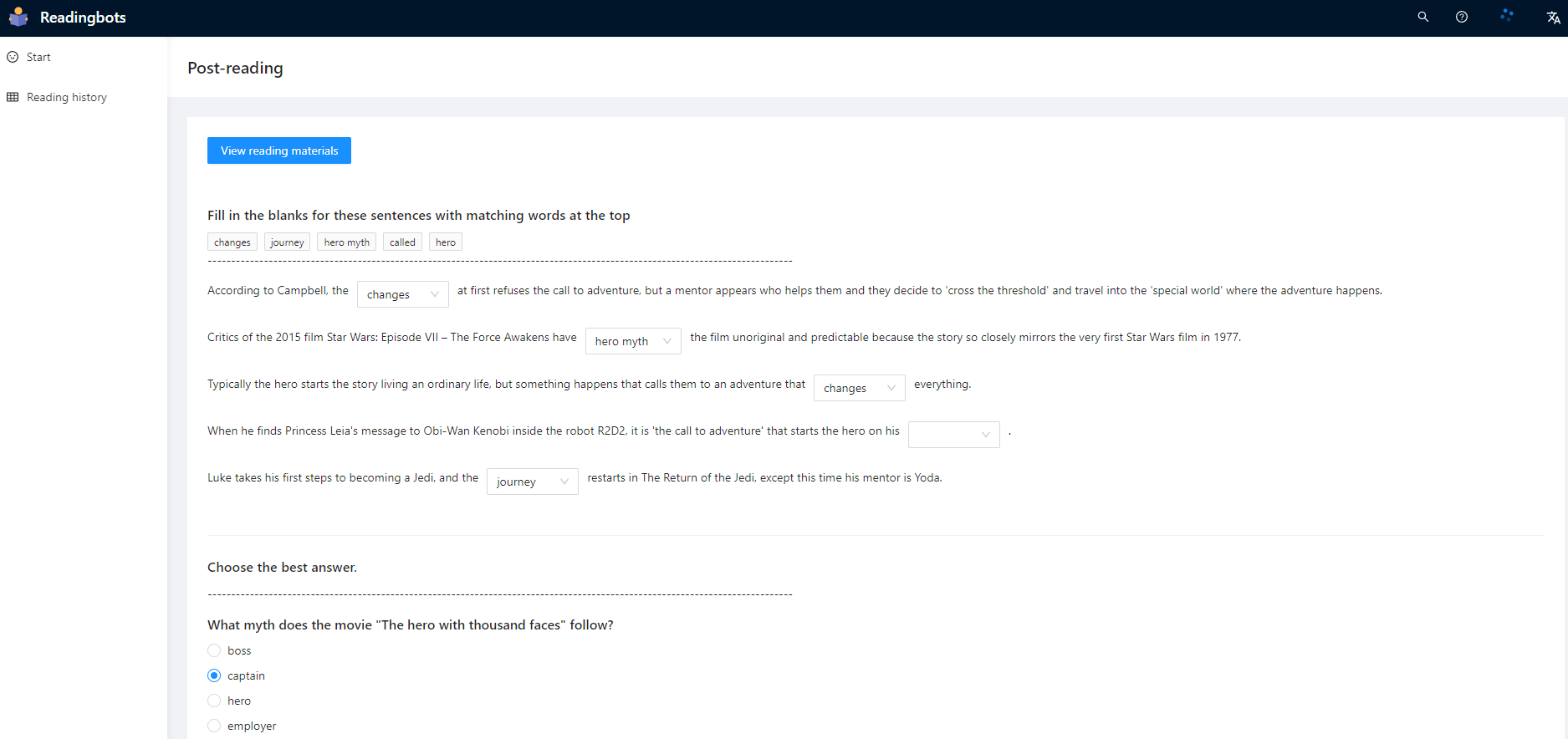


Figure 5 Post-reading page (I)


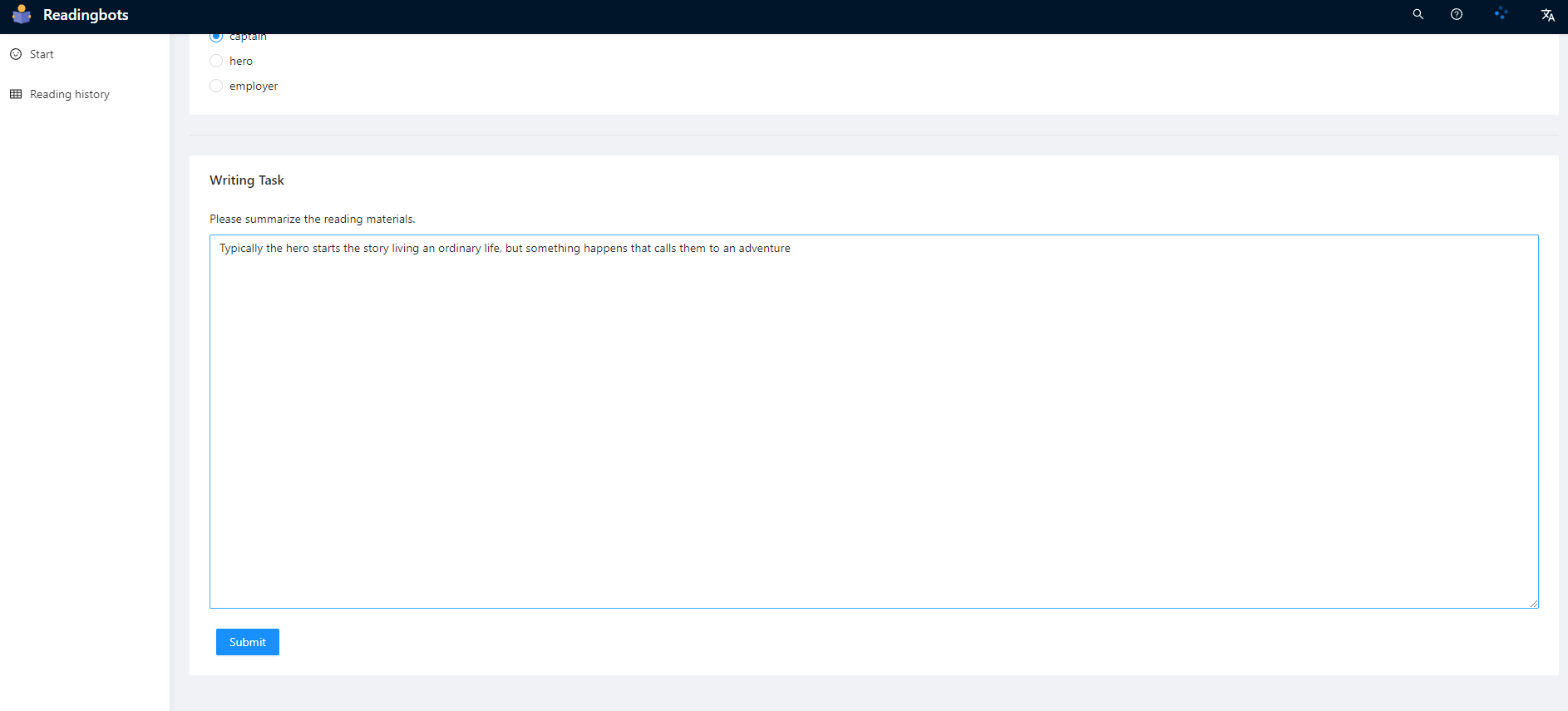


Figure 6 Post-reading page (II)

After submitting the results, readers can view the scores.


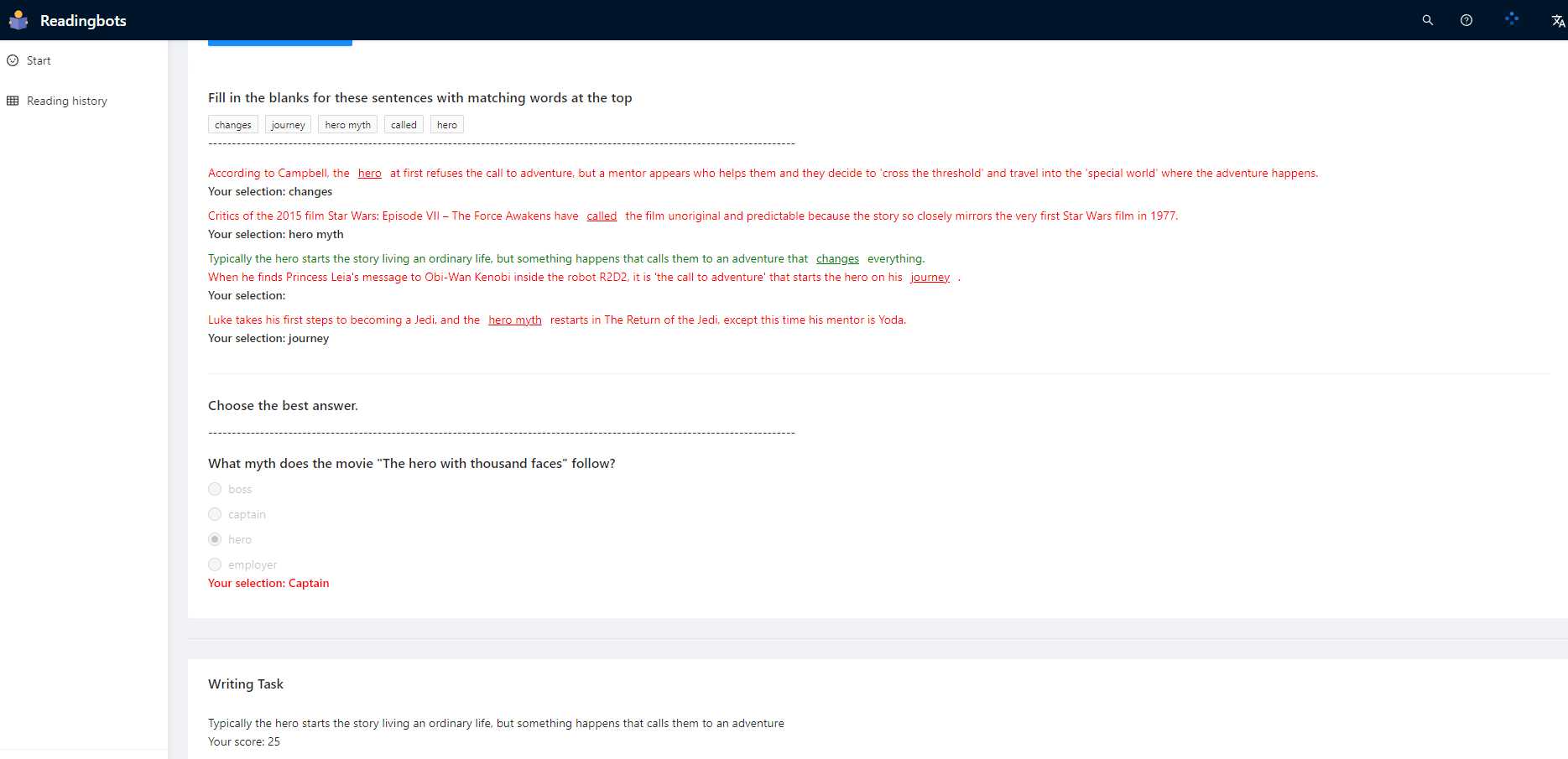


Figure 7 Scoring results page

In the **Reading history** page, readers can view their past reading activities and the recorded details.


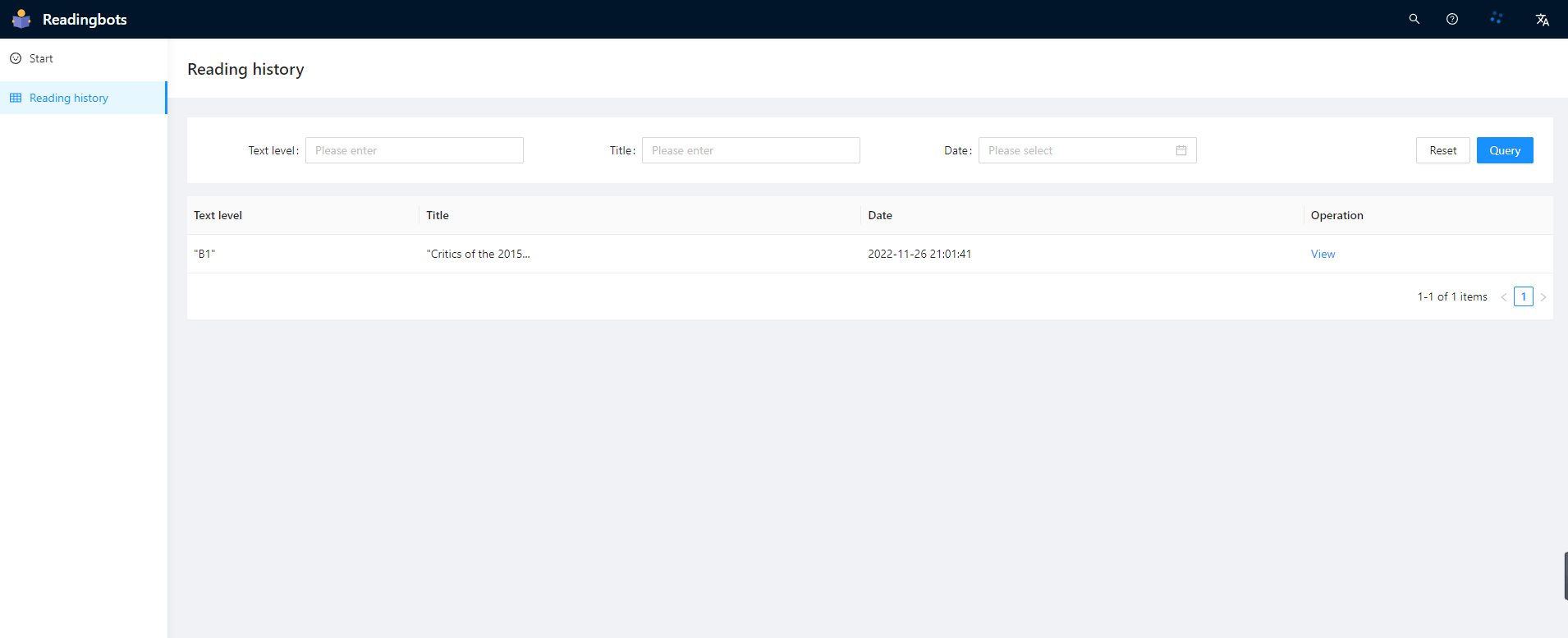


Figure 8 Reading history page


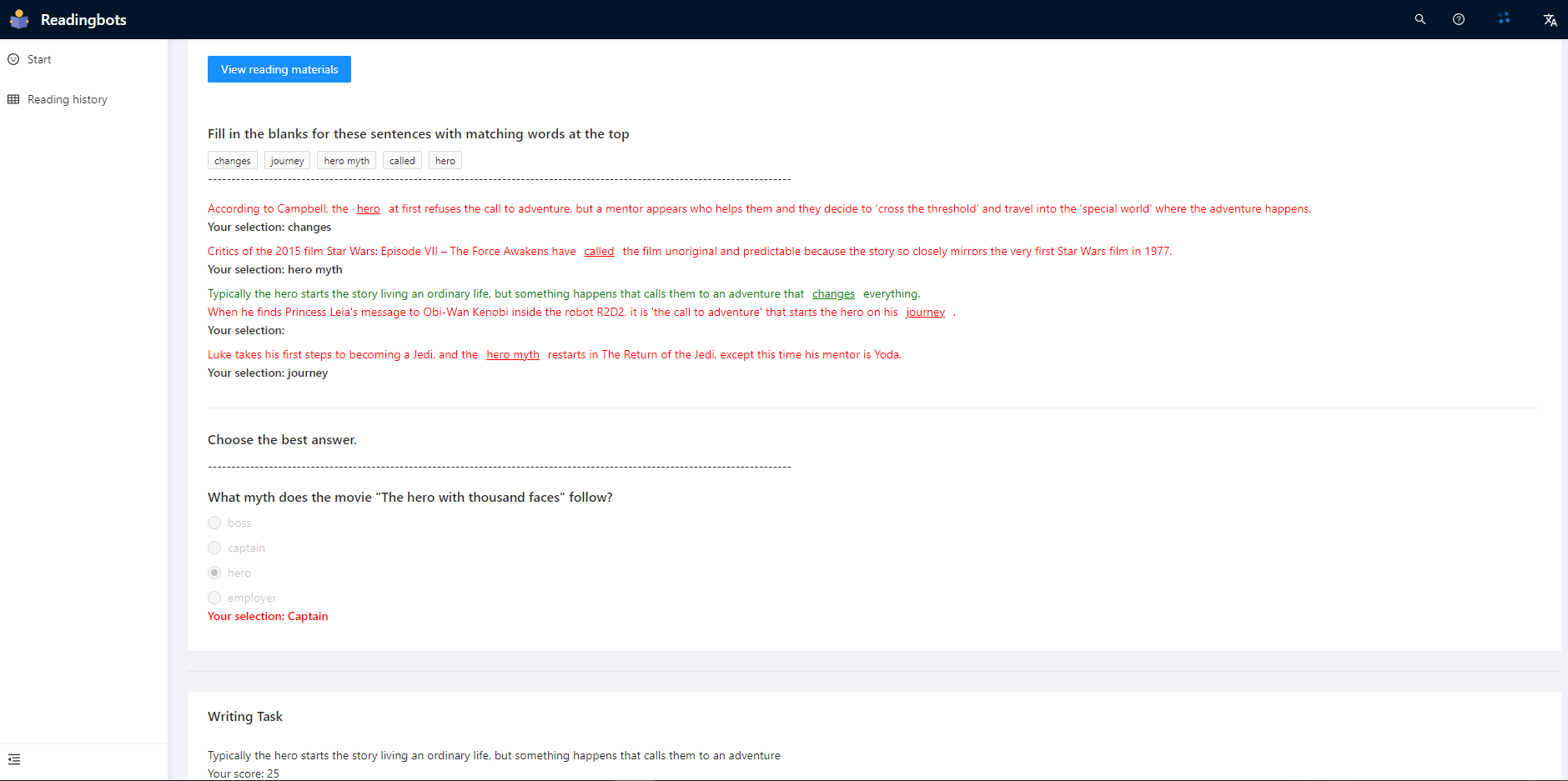


Figure 9 History details page (I)


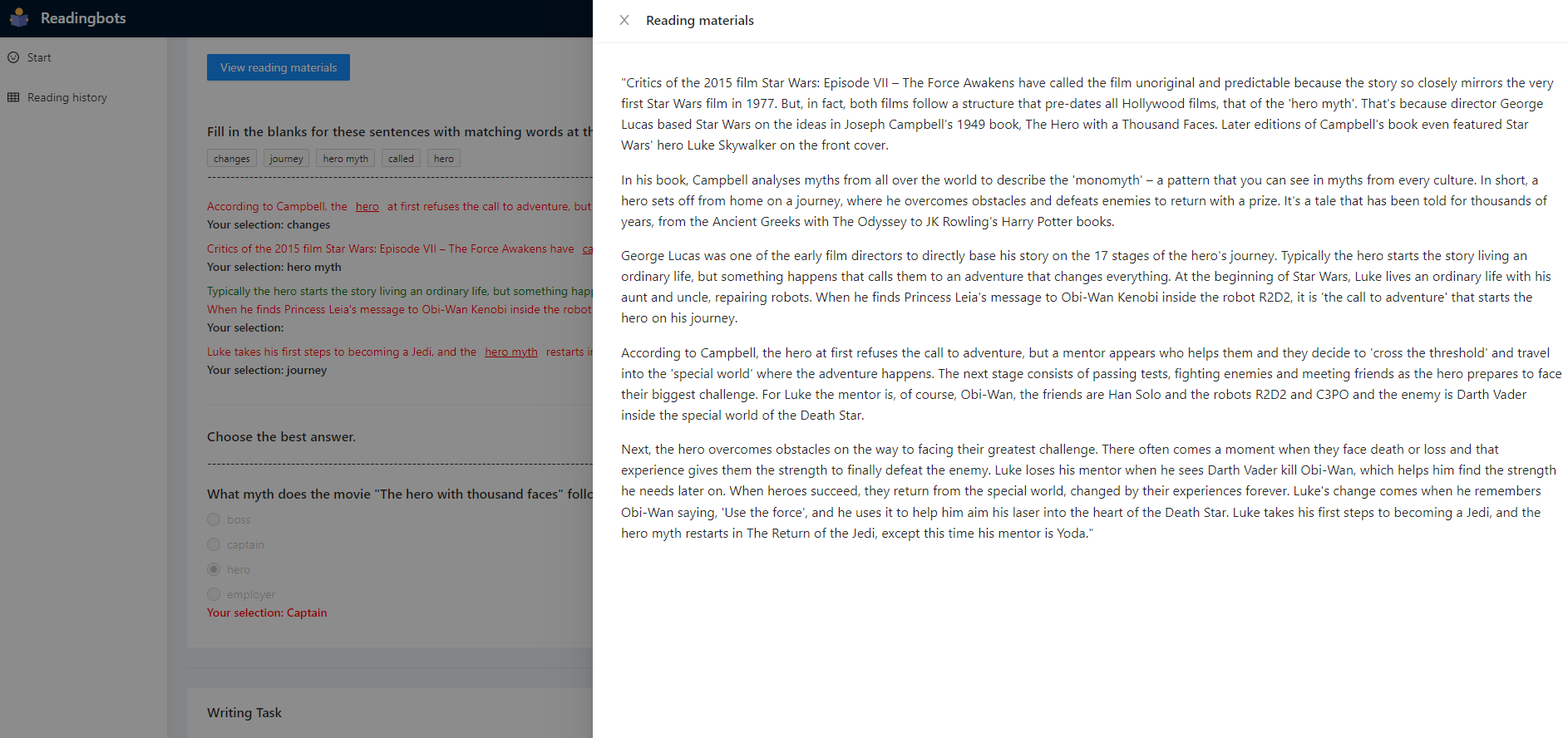


Figure 10 History details page (II)
