# Supplementary material for: Reading bots: The implication of deep learning on guided reading
Source: Front Psychol. 2023 Feb 6;14:980523. doi: 10.3389/fpsyg.2023.980523 (PMC9939479; doi:10.3389/fpsyg.2023.980523)
Supplement: Supplementary file 2 [file Data_Sheet_1.ZIP › readingbots-readingbotsapi.zip!/library_project/books/templates/books/book_list.html]

# All books

{% for book in object\_list %}

- Title: {{ book.title }}
- Subtitle: {{ book.subtitle }}
- Author: {{ book.author }}
- ISBN: {{ book.isbn }}

{% endfor %}
