# Supplementary material for: Reading bots: The implication of deep learning on guided reading
Source: Front Psychol. 2023 Feb 6;14:980523. doi: 10.3389/fpsyg.2023.980523 (PMC9939479; doi:10.3389/fpsyg.2023.980523)
Supplement: Supplementary file 2 [file Data_Sheet_1.ZIP › DiscourseSimplificationService.zip!/DiscourseSimplification-master/DisSim_Demo.pdf]

DisSim: A Discourse-Aware Syntactic Text Simplification Framework for English and German

Christina Niklaus, Matthias Cetto, André Freitas and Siegfried Handschuh

University of St.Gallen

Complex source sentence:

"A fluoroscopic study known as an upper gastrointestinal series is typically the next step in management, although if volvulus is suspected, caution with non water soluble contrast is mandatory as the usage of barium can impede surgical revision and lead to increased post operative complications."

- hard to comprehend by **human readers**
- difficult to analyze by **semantic applications**

APPROACH

Recursive transformation process:

- **35 (English)** and **29 (German)** hand-crafted grammar rules
- Patterns encode **syntactic and lexical features** that can be derived from a sentence's parse tree

RULE	TREGEX PATTERN	EXTRACTED SENTENCE
**SubordinationPostExtractor**	ROOT <<: (S < (NP $.. (VP < +(VP) (**SBAR** <, (*IN* $+ (S < (NP $.. VP)))))))	S < (NP $.. VP).

Each **rule** specifies:

1. How to **split up and rephrase** the input?
2. How to set up a **semantic hierarchy**?

Minimality:
improves the performance of SOTA Open IE approaches in terms of **precision** (up to 346%) and **recall** (up to 52%), i.e. leads to a **lower information loss** and a **higher accuracy** of the extracted tuples.

System	Precision	Recall	AUC
Stanford Open IE	**+346%**	**+52%**	**+597%**
ReVerb	+28%	+40%	+57%
OLLIE	+38%	+8%	+20%
ClausIE	+50%	-20%	+15%
OpenIE-4	+20%	-1%	+3

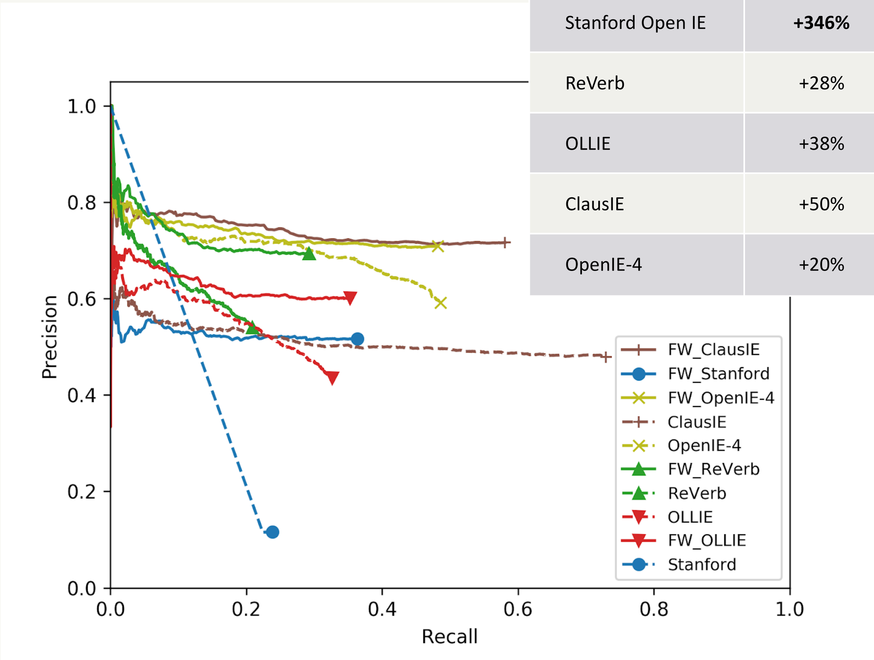

Lightweight semantic representation can be used to **facilitate and improve** a variety of AI tasks.

PROBLEM

APPLICATION

SEMANTIC HIERARCHY

- DisSim breaks down a complex source sentence into a set of **hierarchically ordered** and **semantically interconnected** sentences in the form of **minimal propositions** that present a simplified syntax.
- By taking into account **discourse-level aspects**, we avoid to produce a disconnected sequence of output sentences that are hard to interpret.

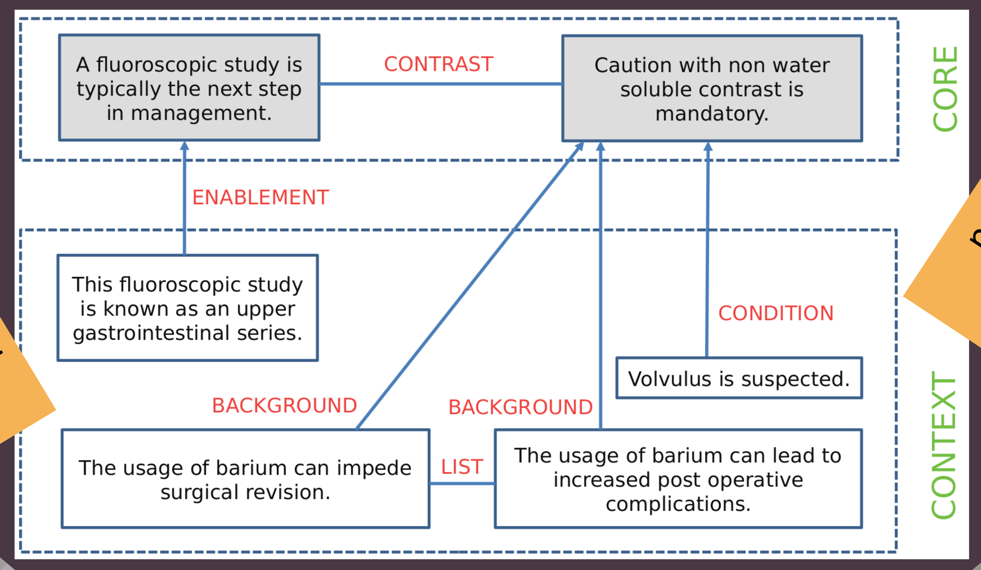

minimal propositions

preservation of coherence structure

METHOD

SUBTASK 1

Split into Minimal **Propositions**

a sequence of **sound, self-contained utterances**, with each of them presenting a **minimal semantic unit** that cannot be further decomposed into meaningful propositions

Simplification rules encode both the **splitting points and rephrasing procedure** for **disembedding clausal and phrasal components** and transforming them into syntactically simplified stand-alone sentences.

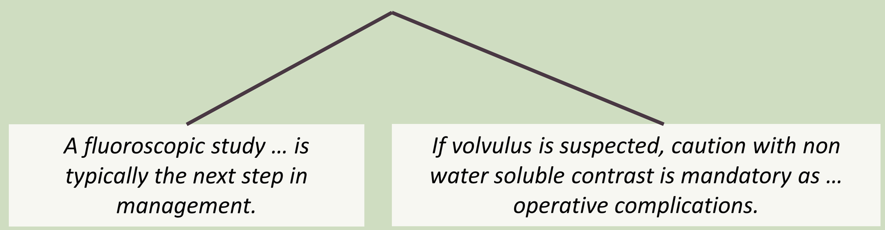

simpler, more **regular structures**

easier to process

loose arrangement of self-contained units that **lack important contextual information**

impedes interpretability

SUBTASK 2

Establish a **Semantic Hierarchy**

1. **Constituency Type Classification**: establishes a **contextual hierarchy** between the split sentences by connecting them with information about their hierarchical level (**core vs. context**) based on the concept of **nuclearity in RST** [1].

hierarchically ordered sentences

2. **Rhetorical Relation Identification**:
   - restores the **semantic relationship** between a pair of split sentences by **identifying and classifying the rhetorical relation** that holds between them.
   - is based on syntactic and lexical features to extract **cue phrases** from the parse tree which are mapped to rhetorical relations using a predefined list of rhetorical cue words [2].

semantically interconnected sentences

"although" -> Contrast

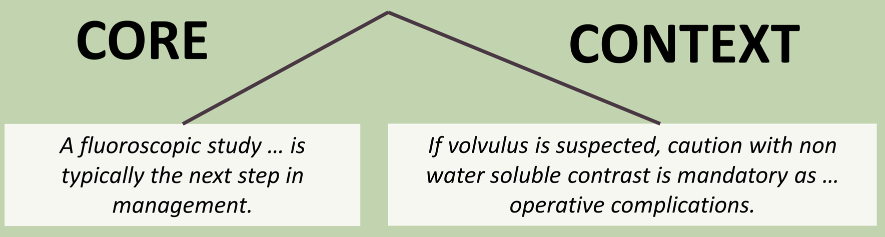

Open IE

Supervised-OIE (alone):
(1) (A fluoroscopic study; known; as an upper gastrointestinal series)
(2) (caution with non water soluble contrast; is; mandatory as the usage of barium)
(3) (as the usage; of barium can impede; surgical revision and lead)
(4) (; to increased; post operative complications)

Supervised-OIE (using DisSim for preprocessing):
(5) #1 0 (A fluoroscopic study; is; typically, the next step in management)
(5a) ELABORATION #2
(5b) CONTRAST #3
(6) #2 1 (This fluoroscopic study; is known; as an upper gastrointestinal series)
(7) #3 0 (Caution with non water soluble; is; mandatory)
(7a) CONTRAST #1
(7b) CONDITION #7
(7c) BACKGROUND #4
(7d) BACKGROUND #5
(7e) BACKGROUND #6
(8) #4 1 (The usage of barium; can impede; surgical revision)
(8a) LIST #5
(8b) LIST #6
(9) #5 1 (The usage of barium; can lead; to increased operative complications)
(9a) LIST #4
(9b) LIST #6
(10) #6 1 (The usage of barium; to increased; post operative complications)
(10a) LIST #4
(10b) LIST #5
(11) #7 1 (Volvulus; is suspected;)

Semantic Hierarchy: enriches the output with important **contextual information** that is needed for a **proper interpretation** of complex assertions.

EXPERIMENTS

SUBTASK 1

DisSim outperforms the SOTA in structural text simplification:

- It generates a **fine-grained output with a high level of grammaticality and meaning preservation**.
- It achieves an improvement of 5%, 4% and 6% with regard to the SAMSA score [3] against the second-best performing approach.
- It can be applied in a **domain independent** manner.
- The full evaluation methodology and detailed results are reported in [4].

SUBTASK 2

A comparative analysis with the annotations contained in the **RST Discourse Treebank** [5] demonstrates that

- DisSim captures the **contextual hierarchy** between the split sentences with a **precision of 90%**, and
- DisSim reaches an **average precision of 70%** for the **classification of the rhetorical relations** that hold between them.

Leaf nodes are recursively simplified in a top-down fashion until no more rule matches.

logical structure of utterances that present a **simplified syntax**

OUTPUT

Christina Niklaus
christina.niklaus@unisg.ch

Institute of Computer Science
University of St. Gallen

References

[1] Mann and Thompson, 1988. Rhetorical Structure Theory: Toward a Functional Theory of Text Organization.
[2] Taboada and Das, 2013. Annotation upon Annotation: Adding Signalling Information to a Corpus of Discourse Relations.
[3] Sulem et al., 2018. Semantic Structural Evaluation for Text Simplification.
[4] Niklaus et al., 2019. Transforming Complex Sentences into a Semantic Hierarchy.
[5] Carlson et al., 2002. RST Discourse Treebank.
