# Supplementary material for: Reading bots: The implication of deep learning on guided reading
Source: Front Psychol. 2023 Feb 6;14:980523. doi: 10.3389/fpsyg.2023.980523 (PMC9939479; doi:10.3389/fpsyg.2023.980523)
Supplement: Supplementary file 2 [file Data_Sheet_1.ZIP › DiscourseSimplificationService.zip!/DiscourseSimplification-master/supplemental_material/discourse_tree_example_volvulus.pdf]

Step-by-Step Example for the Discourse Tree Creation

March 2021

In the following, a step-by-step example is given, illustrating the transformation stage on the following complex source sentence:

> *"A fluoroscopic study which is known as an upper gastrointestinal series is typically the next step in management, although if volvulus is suspected, caution with non water soluble contrast is mandatory as the usage of barium can impede surgical revision and lead to increased post operative complications."*

With the help of the 35 hand-crafted grammar rules, the input is recursively transformed into a discourse tree, i.e. a set of hierarchically ordered and semantically interconnected sentences that present a simplified syntax.

(1) Initialization of the discourse tree:

A fluoroscopic study which is known as an upper gastrointestinal series is typically the next step in management, although if volvulus is suspected, caution with non water soluble contrast is mandatory as the usage of barium can impede surgical revision and lead to increased post operative complications.

(2) Rule application on the marked leaf:

A fluoroscopic study which is known as an upper gastrointestinal series is typically the next step in management, although if volvulus is suspected, caution with non water soluble contrast is mandatory as the usage of barium can impede surgical revision and lead to increased post operative complications.

(3) Result of the rule application:

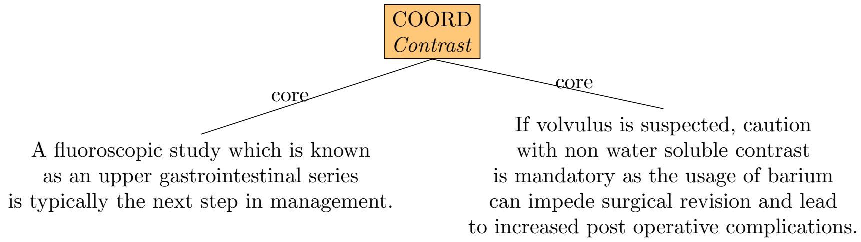

(4) Rule application on the marked leaf:

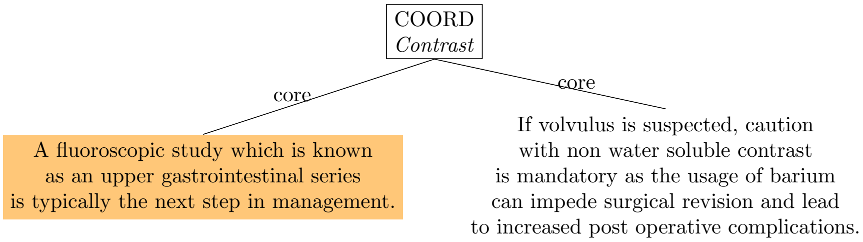

(5) Result of the rule application:

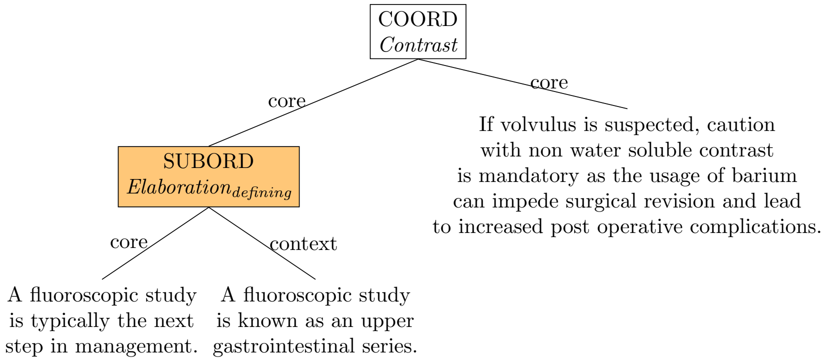

(6) Rule application on the marked leaf:

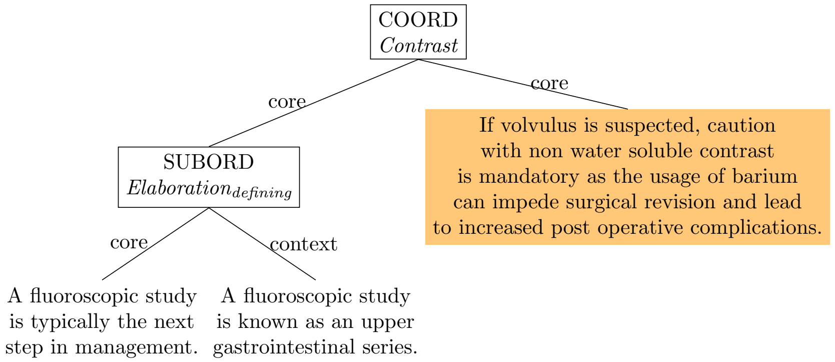

(7) Result of the rule application

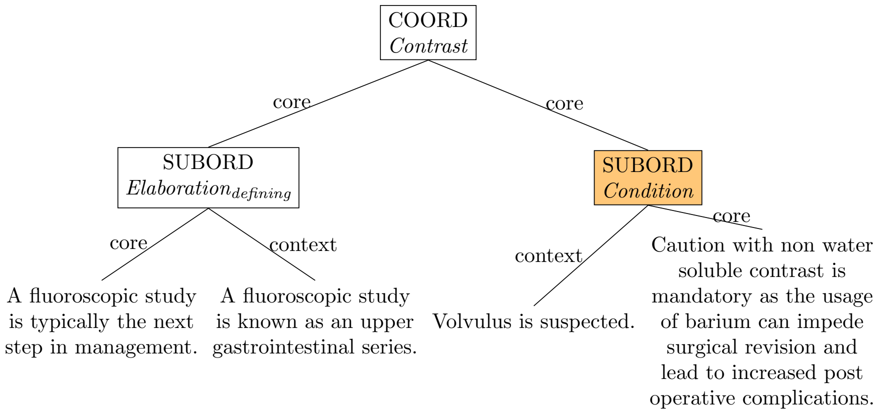

(8) Rule application on the marked leaf:

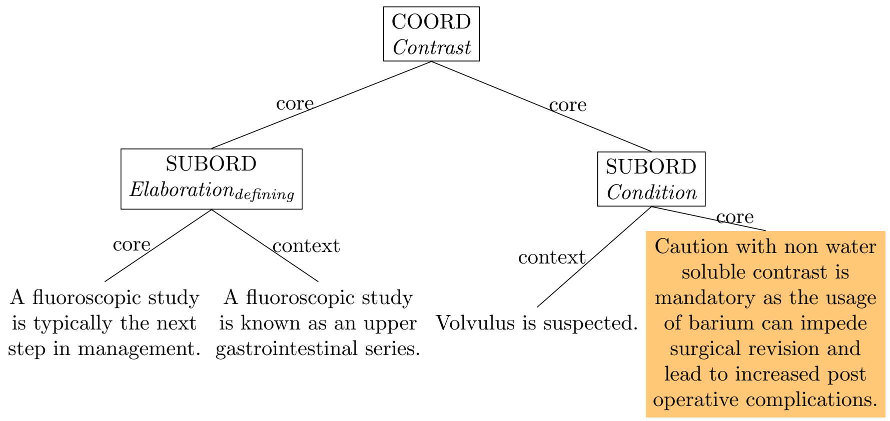

(9) Result of the rule application:

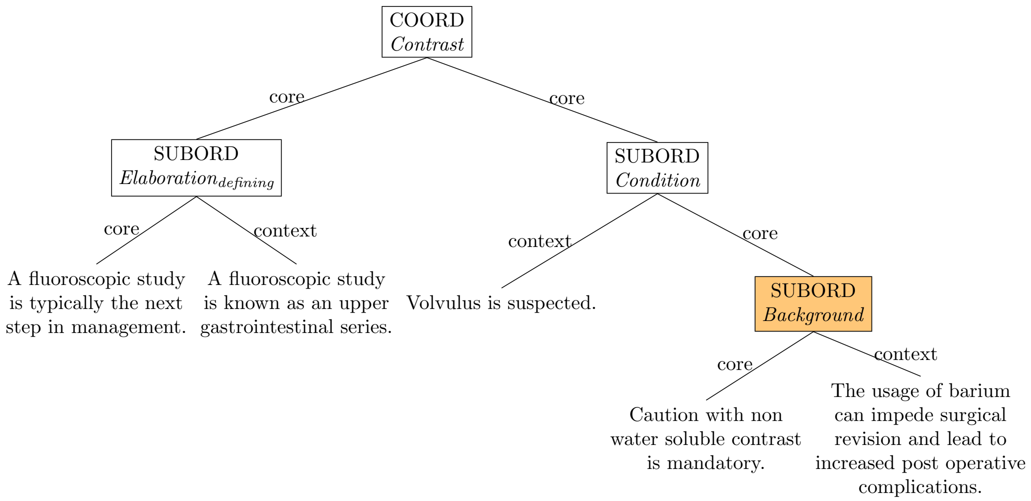

(10) Rule application on the marked leaf:

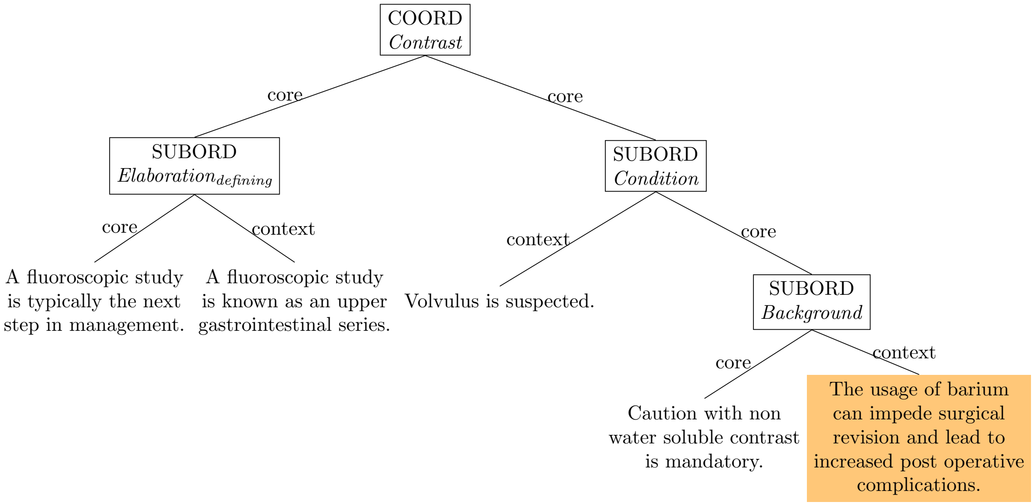

(11) Result of the rule application:

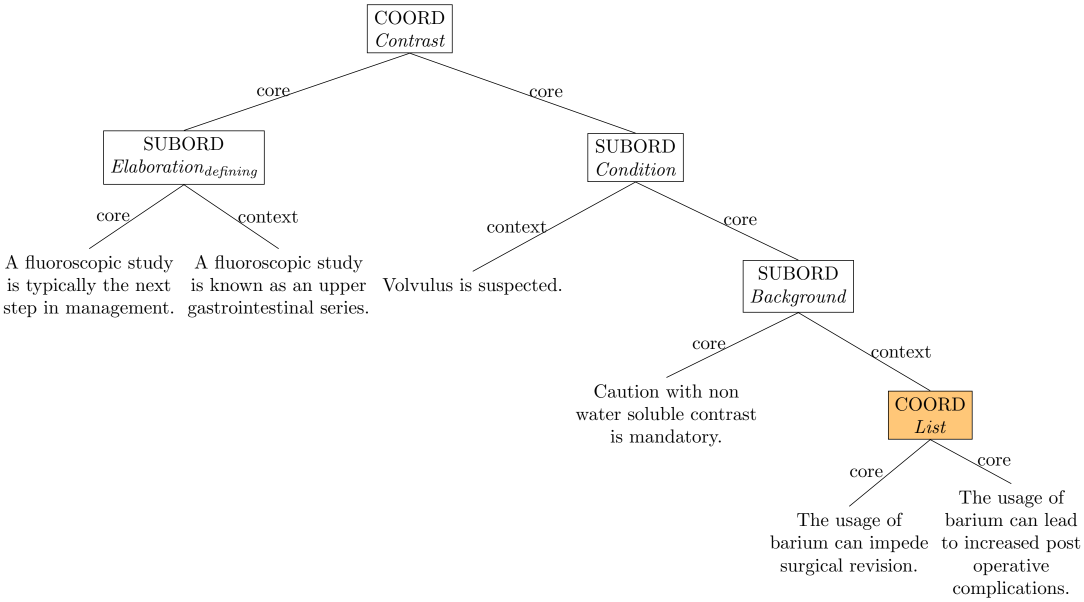

Figure 1: Final discourse tree of the example sentence.

(12) STOP. No more transformation rule pattern matches.

When no more transformation pattern matches, the algorithm stops. The final discourse tree for our running example is shown in Figure 1.
