# Supplementary material for: Reading bots: The implication of deep learning on guided reading
Source: Front Psychol. 2023 Feb 6;14:980523. doi: 10.3389/fpsyg.2023.980523 (PMC9939479; doi:10.3389/fpsyg.2023.980523)
Supplement: Supplementary file 2 [file Data_Sheet_1.ZIP › DiscourseSimplificationService.zip!/DiscourseSimplification-master/supplemental_material/Step_by_Step_Example.pdf]

A Step-by-Step Example for the Discourse Tree Creation

May 2019

In the following, a step-by-step example is given, illustrating the transformation stage where the input text is recursively transformed into a set of syntactically simplified sentences in a top-down fashion.

(1) Initialization of the discourse tree:

Although the Treasury will announce details of the November refunding on Monday,
the funding will be delayed if Congress and President Bush
fail to increase the Treasury's borrowing capacity.

(2) Rule application on the marked leaf:

Although the Treasury will announce details of the November refunding on Monday,
the funding will be delayed if Congress and President Bush
fail to increase the Treasury's borrowing capacity.

(3) Result of the rule application:

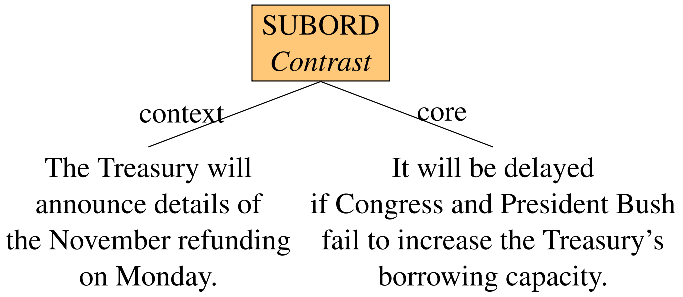

(4) Rule application on the marked leaf:

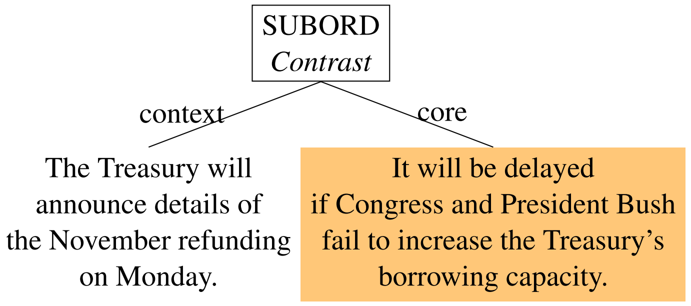

(5) Result of the rule application:

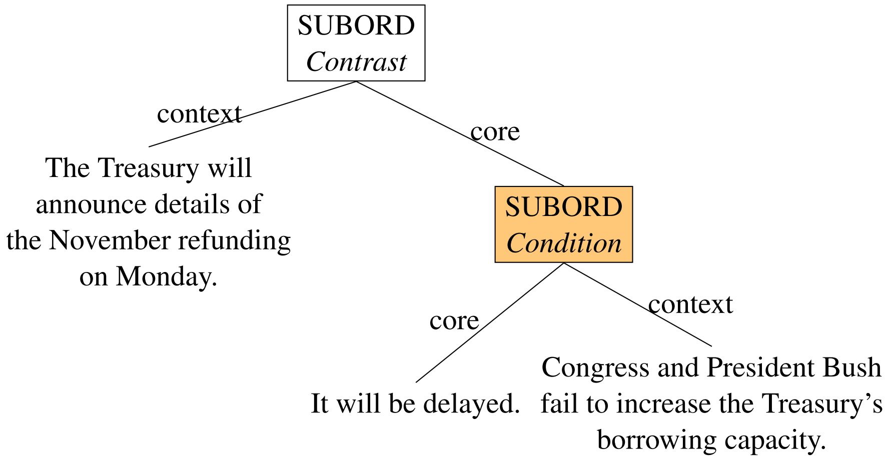

(6) Rule application on the marked leaf:

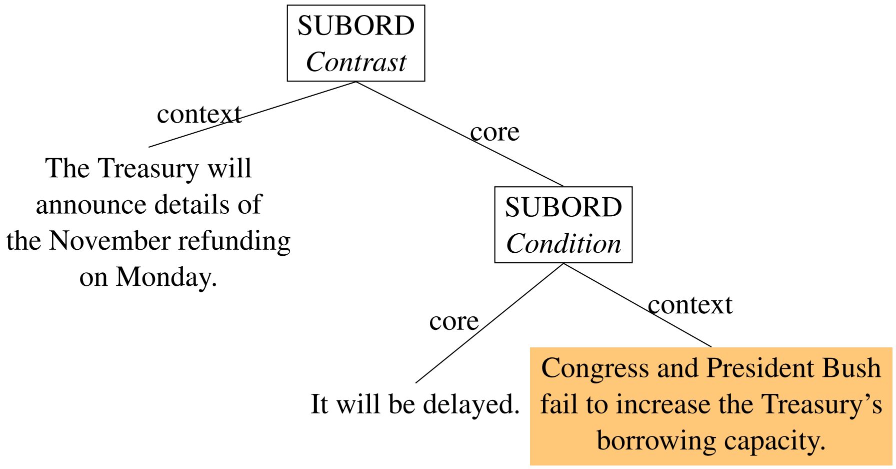

(7) Result of the rule application

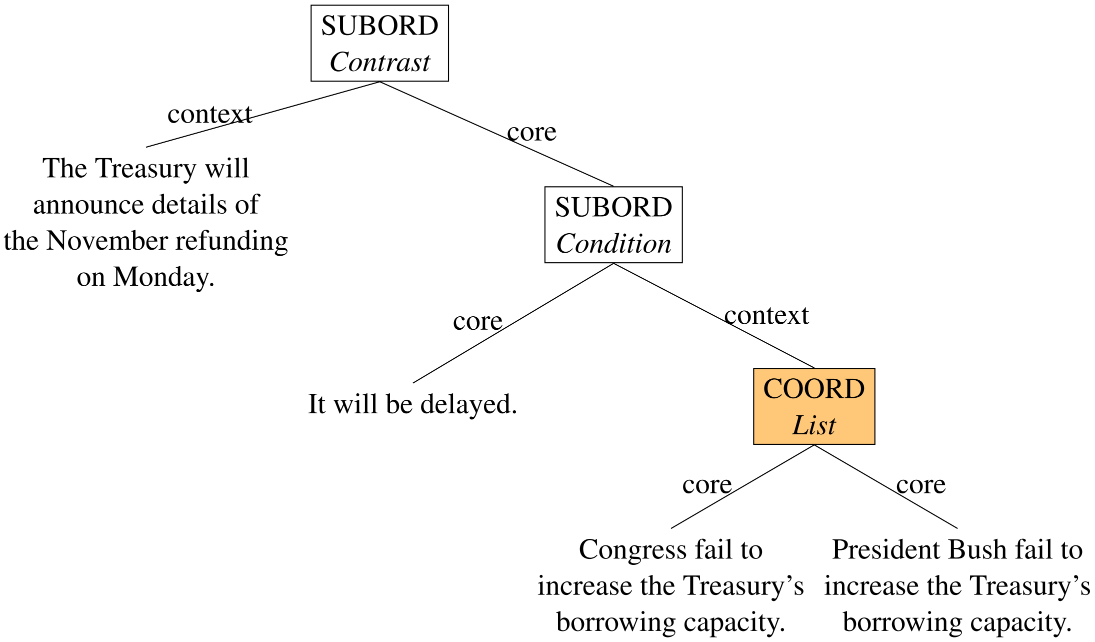

(8) Rule application on the marked leaf:

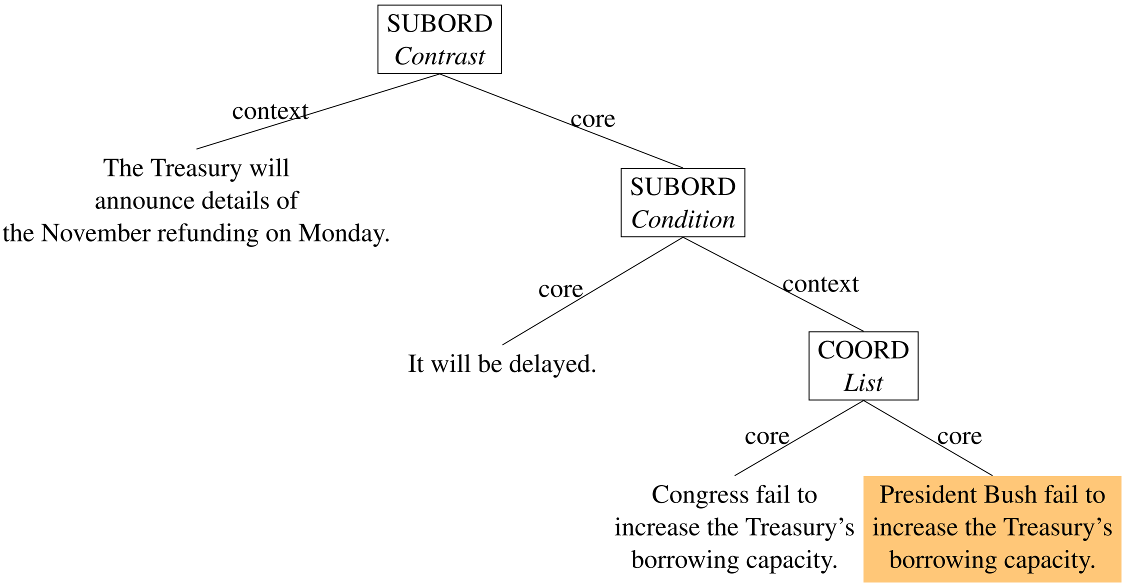

(9) Result of the rule application:

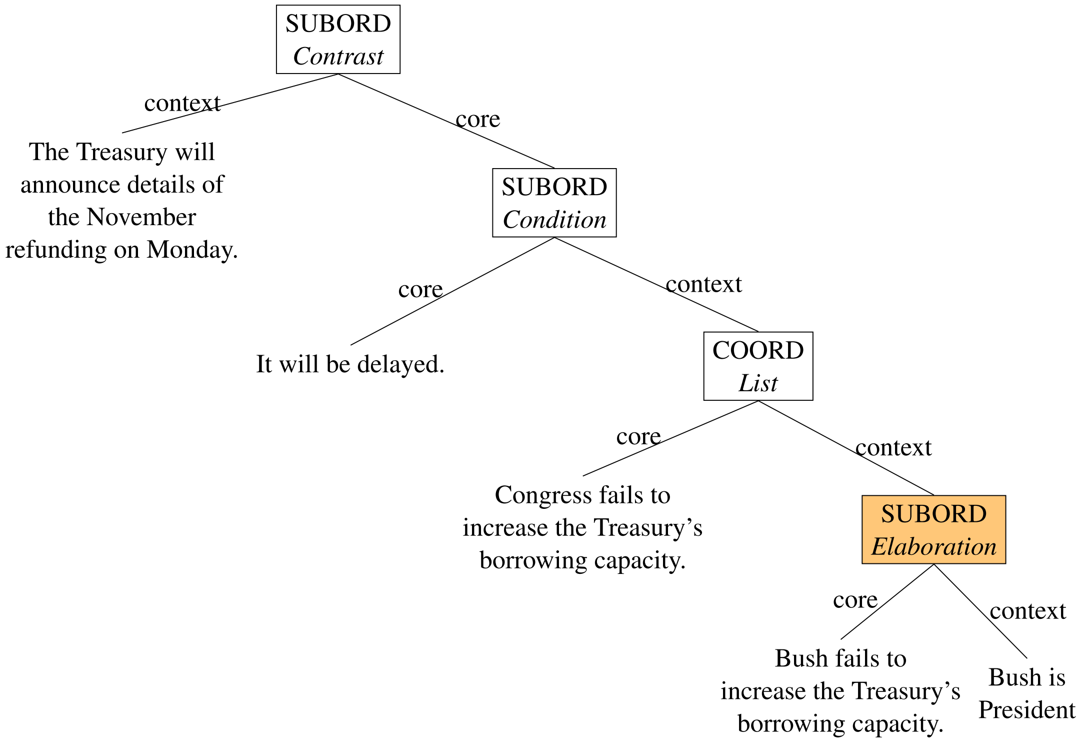

(10) Rule application on the marked leaf:

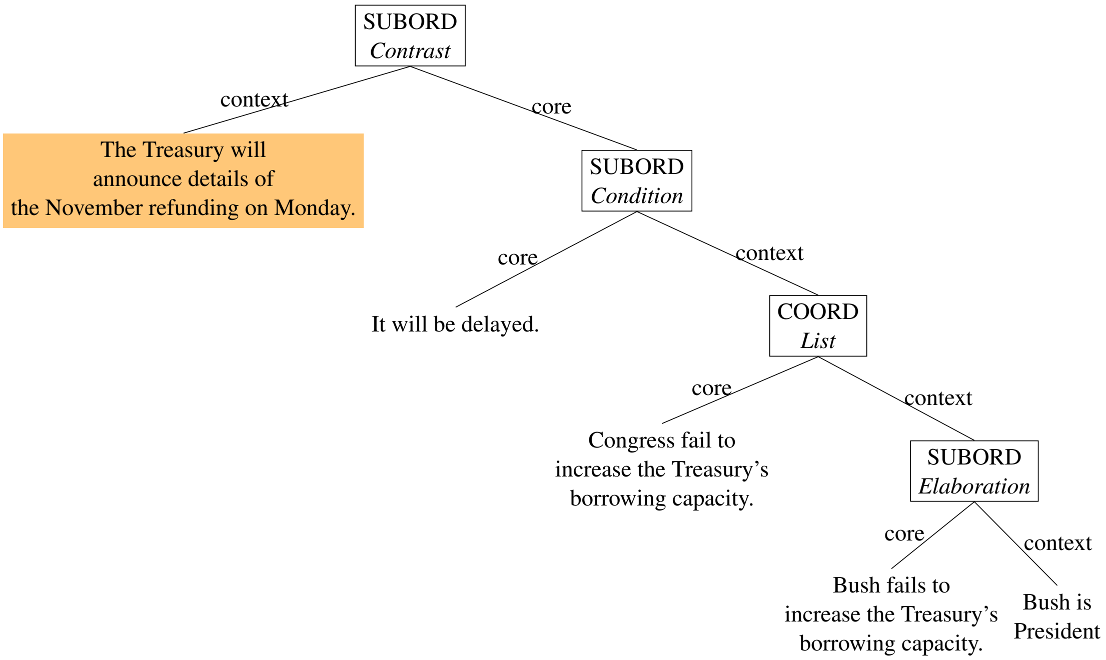

(11) Result of the rule application:

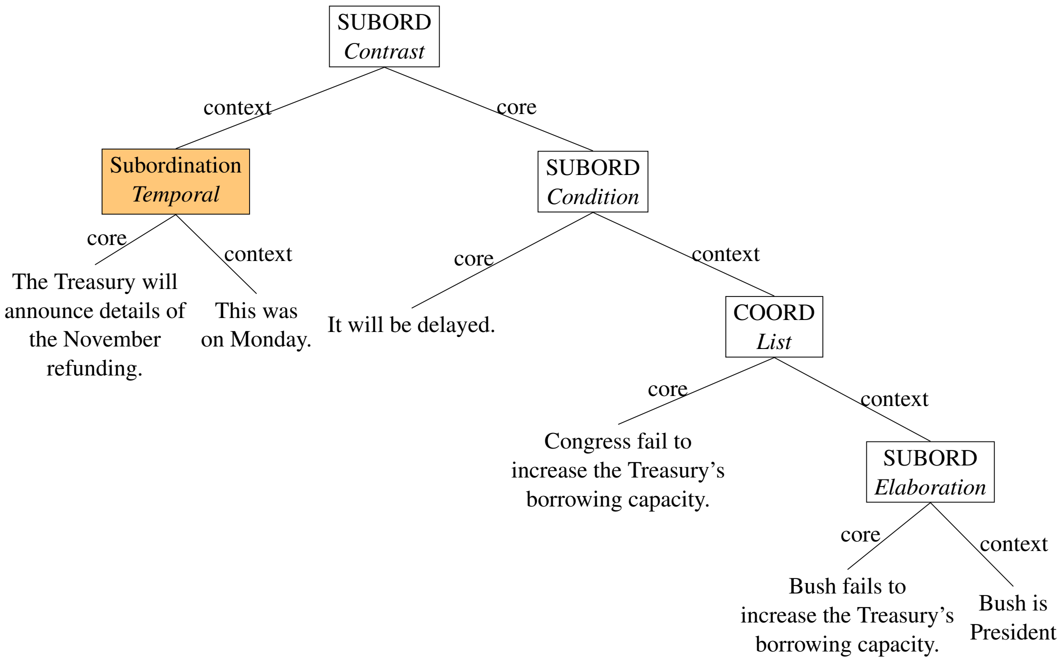

(12) STOP. No more transformation rule pattern matches.
