# Supplementary material for: Reading bots: The implication of deep learning on guided reading
Source: Front Psychol. 2023 Feb 6;14:980523. doi: 10.3389/fpsyg.2023.980523 (PMC9939479; doi:10.3389/fpsyg.2023.980523)
Supplement: Supplementary file 2 [file Data_Sheet_1.ZIP › DiscourseSimplificationService.zip!/DiscourseSimplification-master/supplemental_material/Transformation_Patterns.pdf]

# Transformation Patterns

May 2019

## 1 Clausal Disembedding

### 1.1 Coordinate Clauses

| Rule | Tregex pattern | Extracted sentence |
|---|---|---|
| CoordinationExtractor | ROOT <<: (S < (S $.. [S])) | [S]. |
| Example: The Assistant Attorney in Orlando investigated the modeling company, *and decided that they were not doing anything wrong, and after Pearlman's bankruptcy, the company emerged unscathed and was sold to a Canadian company.* | | |

Table 1: The transformation rule pattern for splitting coordinate clauses. The framed pattern represents the part of a sentence that is extracted from the source and turned into a stand-alone sentence.

### 1.2 Adverbial Clauses

### 1.3 Relative Clauses

### 1.4 Reported Speech

## 2 Phrasal Disembedding

### 2.1 Coordinate Verb Phrases

### 2.2 Coordinate Noun Phrases

### 2.3 Appositions

### 2.4 Prepositional Phrases

### 2.5 Adjectival and Adverbial Phrases

### 2.6 Lead Noun Phrases

<table>
<tr><th>Rule</th><th>Tregex pattern</th><th>Extracted sentence</th></tr>
<tr><td>Subordination-PostExtractor</td><td>ROOT <<: (S < (NP $.. (VP < +(VP) (SBAR < ( S < (NP $.. VP ))))))</td><td>S < (NP $.. VP .</td></tr>
<tr><td colspan="3">Example: It is also used by those who commute into the city using Metrorail, as the two systems are connected at the Government Center and Brickell stations.</td></tr>
<tr><td>Subordination-PreExtractor</td><td>ROOT <<: (S < (SBAR < ( S < (NP $.. VP) ) $.. (NP $.. VP)))</td><td>S < (NP $.. VP) .</td></tr>
<tr><td colspan="3">Example: Though shorts are an option for many casual occasions, they may also be inappropriate for more formal occasions, and a skirt or dress, such as a sundress, may be the best choice.</td></tr>
<tr><td>PurposePost-Extractor</td><td>ROOT <<: (S < (NP $.. (VP < +(VP) (NP|PP $.. ( S <<, (VP <<, /(T|t)o/) )))))</td><td>This + Be + S <<, (VP <<, /(T|t)o/) .</td></tr>
<tr><td colspan="3">Example: In 1923 he joined Ireland's new Department of External Affairs and was sent to Geneva in 1929 to act as Ireland's representative to the League of Nations.</td></tr>
<tr><td>PurposePre-Extractor</td><td>ROOT <<: (S < ( S <<, (VP <<, /(T|t)o/) $.. (NP $.. VP)))</td><td>This + Be + S <<, (VP <<, /(T|t)o/) .</td></tr>
<tr><td colspan="3">Example: To support this claim, he points out the actor is wearing a very large fake moustache and a hat, and when his moustache detached before the end of the film, he hid his face by an arm until the moustache was reattached.</td></tr>
<tr><td>Subordination-PostPurpose-Extractor</td><td>ROOT <<: (S < (NP $.. (VP < +(VP) (SBAR < ( S <<, (VP <<, /(T|t)o/) )))))</td><td>This + Be + S <<, (VP <<, /(T|t)o/) .</td></tr>
<tr><td colspan="3">Example: A graduate student, Ian Gray, is researching the evolution of human eyes with Karen and Kenny, in order to discredit creationists by proving that eyes have evolved.</td></tr>
<tr><td>Subordination-PrePurpose-Extractor</td><td>ROOT <<: (S < (SBAR < ( S <<, (VP <<, /(T|t)o/) ) $.. (NP $.. VP)))</td><td>This + Be + S <<, (VP <<, /(T|t)o/) .</td></tr>
<tr><td colspan="3">Example: In order to cater for large items and fast loading, the entire tail section was hinged, and could be opened using hydraulic actuators.</td></tr>
</table>

Table 2: A selection of transformation rule patterns. A framed pattern represents the extracted part of a sentence. An underlined pattern refers to its referent. A pattern in bold will be excluded in the remaining sentence.

<table>
<tr><th>Rule</th><th>Tregex pattern</th><th>Extracted sentence</th></tr>
<tr><td>Non-Restrictive-Relative-ClausePrep-WhichWho-Extractor</td><td>ROOT <<: (S << (NP <, NP & < (/,/ $+ (**SBAR** <, (**WHPP** $+ S & <, IN=in & < – WHNP=whnp) & ?$+ /,/))))</td><td>*in + attachment phrase + whnp.*</td></tr>
<tr><td colspan="3">Example: His family had been exiled to, and enrolled into Harvard University, *at which he studied archaeology.*</td></tr>
<tr><td>Non-Restrictive-Relative-ClauseWhere-Extractor</td><td>ROOT <<: (S << (/.*/ < (NP|PP $+ (/,/ $+ (**SBAR** <, (**WHADVP** $+ S & <<: WRB) & ?$+ /,/)))))</td><td>*Extraction.*</td></tr>
<tr><td colspan="3">Example: The noted communist, Sakhavu Kurumpakara Thankappan, was born and raised in Kurumpakara, *where a memorial dedicated to him is situated at the Udayonmuttam Junction.*</td></tr>
<tr><td>Non-Restrictive-Relative-ClauseWhom-Extractor</td><td>ROOT <<: (S << (NP <, NP & < (/,/ $+ (**SBAR** <, (**WHNP** $+ (S <, NP & < – (VP ? < +(VP) PP=pp)) & <<: (WP <: whom)) & ?$+ /,/))))</td><td>*np + vp + attachment phrase + pp.*</td></tr>
<tr><td colspan="3">Example: He is best known for his work with The Byrds, *whom he joined in September 1968 as a replacement for the band’s original bass player Chris Hillman.*</td></tr>
<tr><td>Non-Restrictive-Relative-ClauseWhose-Extractor</td><td>ROOT <<: (S << (NP < (NP $+ (/,/ $+ (**SBAR** <, (**WHNP** $+ S & <, (/WP \\$/ $+ /.*/)) & ?$+ /,/)))))</td><td>*Attachment phrase +* ”’s” *+ extraction.*</td></tr>
<tr><td colspan="3">Example: Lacking a close relationship with his own estranged daughter, Dunn comes to establish a strong bond with Maggie, *whose own family cares little for her well-being.*</td></tr>
<tr><td>Non-Restrictive-Relative-Clause-WhoWhich-Extractor</td><td>ROOT <<: (S << (NP <, NP & < (/,/ $+ (**SBAR** <, (**WHNP** $+ S & <<: WP|WDT) & ?$+ /,/))))</td><td>*Attachment phrase + extraction.*</td></tr>
<tr><td colspan="3">Example: However, while there, she discovered she could live on her income as a novelist and also met her husband, *who was completing his doctorate in physics.*</td></tr>
</table>

Table 3: A selection of transformation rule patterns. A framed pattern represents the extracted part of a sentence. An underlined pattern refers to its referent. A pattern in bold will be excluded in the remaining sentence.

<table>
<tr><th>Rule</th><th>Tregex pattern</th><th>Extracted sentence</th></tr>
<tr><td>Participial-Middle-Extractor</td><td>ROOT <<: (S < (VP< +(VP) /VB.*/) & << (NP|PP <, (NP ?$+ PP & $ + + (/,/ $+ (VP [<, (ADVP|PP $+ VBG|VBN) | <, VBG|VBN] & ?$+ /,/)))))</td><td>Attachment phrase + Be + extraction.</td></tr>
<tr><td colspan="3">Example: The Metox, named after its manufacturer, was a high frequency very sensitive radar receiver manufactured by a small French company in occupied Paris, which could detect ASV transmissions from patrolling Allied aircraft.</td></tr>
<tr><td>Shared-NPPost-Participial-Extractor</td><td>String participalNode = "(__ [== S=s | == (PP|ADVP < +(PP|ADVP) S=s)]) : (=s <: (VP <<, VBG|VBN))"; ROOT <<: (S < (NP $.. (VP < +(VP) (NP|PP $.. " + participalNode + "))))</td><td>Attachment phrase + (Have) + Be + extraction.</td></tr>
<tr><td colspan="3">Example: He served as chief judge from 1987 to 1994, assuming senior status on November 2, 1995.</td></tr>
<tr><td>Shared-NPPre-Participial-Extractor</td><td>String participalNode = "(__=node [== S=s | == (PP|ADVP < +(PP|ADVP) S=s)]) : (=s <: (VP <<, VBG|VBN))";<br>ROOT <<: (S < " + participalNode + ") : (=node $.. (NP $.. VP))</td><td>Attachment phrase + (Have) + Be + extraction.</td></tr>
<tr><td colspan="3">Example: Founded in 1973, the campus is situated on a hillside below the neighborhood.</td></tr>
</table>

Table 4: A selection of transformation rule patterns. A framed pattern represents the extracted part of a sentence. An underlined pattern refers to its referent. A pattern in bold will be excluded in the remaining sentence.

<table>
<tr><th>RULE</th><th>TREGEX PATTERN</th><th>EXTRACTED SENTENCE</th></tr>
<tr><td>Restrictive-Relative-ClauseWhom-Extractor</td><td>ROOT <<: (S << (NP <, <u>NP</u> & < (SBAR <, (WHNP $+ (S <, NP=np & < − (VP=vp ? < +(VP) PP=pp)) & <<: (WP <: whom)))))</td><td>np + vp + attachment phrase + pp.</td></tr>
<tr><td colspan="3">EXAMPLE:</td></tr>
<tr><td>Restrictive-Relative-ClauseWhose-Extractor</td><td>ROOT <<: (S << (NP < (<u>NP</u> $+ (SBAR <, (WHNP $+ S & <, (/ WP\\$/ $+ /.*/))))))</td><td>Attachment phrase + ”’s” + extraction.</td></tr>
<tr><td colspan="3">EXAMPLE: The rescue operation to reach Flight 608 was carried out by <u>the Canadian Forces</u> whose plane spotted the downed aircraft and parachuted in nine search-rescue workers.</td></tr>
<tr><td>Restrictive-Relative-Clause-WhoWhich-Extractor</td><td>ROOT <<: (S << (NP <, (<u>NP</u> $ + + (SBAR <, (WHNP $+ S & <<: WP|WDT) & ?$+ /,/))))</td><td>Attachment phrase + extraction.</td></tr>
<tr><td colspan="3">EXAMPLE: Ishak Belfodil is a <u>Franco-Algerian football player</u> who currently plays for French club Olympique Lyonnais in Ligue 1.</td></tr>
<tr><td>Restrictive-Relative-Clause-Without-Relative-Pronoun-Extractor</td><td>ROOT <<: (S << (NP <, (<u>NP</u> $ + + (SBAR <: (S < (VP ? < (PP ? <: IN)))))))</td><td>Attachment phrase + extraction.</td></tr>
<tr><td colspan="3">EXAMPLE: In 1894 Verga moved back to <u>the house</u> he was born in, where he died of a cerebral thrombosis in 1922.</td></tr>
<tr><td>Restrictive-Participial-Extractor</td><td>ROOT <<: (S < (VP < +(VP) /.*/=mainverb) & << (NP|PP <, (<u>NP</u> $+ (VP [<, (ADVP|PP $+ VBG|VBN) | <, VBG|VBN] )) & [> (PP ! > S)| > (VP > S)]))</td><td>Attachment phrase + BE + extraction.</td></tr>
<tr><td colspan="3">EXAMPLE: The Muppets at Walt Disney World is <u>a film</u> starring Jim Henson’s Muppets at Walt Disney World, and this was the last Muppets project that Jim Henson worked on before his death in 1990.</td></tr>
</table>

Table 5: A selection of transformation rule patterns. A framed pattern represents the extracted part of a sentence. An underlined pattern refers to its referent. A pattern in bold will be excluded in the remaining sentence.

| Rule | Tregex pattern | Extracted sentence |
|---|---|---|
| PreAttribution-Extractor | ROOT <<: (S < (S\|SBAR\|SBARQ $.. (NP=np [$,, VP=vp \| $.. VP=vp]))); *vb* is an attribution-verb; Extraction ← text until *np* and text after *vp* | *This* + Be + *what extraction.* |
| Example: Witness memories don't get better with time, *she said in an interview with the International Herald Tribune.* | | |
| Quoted-Attribution-PostExtractor | ROOT <<: (S < (NP $.. (VP < +(VP) (SBAR [,, /“/=start \| <<, /“/=start] [.. /”/=end \| << − /”/=end])))); Extraction ← text until *start* and text after *end* | *This* + Be + *what extraction.* |
| Example: *Pauli remarked sadly* ”It is not even wrong”. | | |
| Quoted-Attribution-PreExtractor | ROOT <<: (S < (S\|SBAR\|SBARQ [,, /“/=start \| <<, /“/=start] [.. /”/=end \| << − /”/=end] $.. (NP [$,, VP \| $.. VP]))); Extraction ← text until *start* and text after *end* | *This* + Be + *what extraction.* |
| Example: | | |
| Subordination-PostAttribution-Extractor | ROOT <<: (S < (NP $.. (VP=vp < +(VP) SBAR=sbar))); *vb* is an attribution-verb; Extraction ← text until *sbar* and text after *sbar* | *This* + Be + *what extraction.* |
| Example: *Later, Ellis claimed that* the character was not in fact based on his father. | | |

Table 6: A selection of transformation rule patterns. A framed pattern represents the extracted part of a sentence. An underlined pattern refers to its referent. A pattern in bold will be excluded in the remaining sentence.

| Rule | Tregex pattern | Extracted sentence |
|---|---|---|
| SharedNPPost-Coordination-Extractor | ROOT <<: (S < (NP $.. (VP < +(VP) (VP > VP $.. VP )))) | NP + VP . |
| Example: After Pearlman's bankruptcy, the company emerged unscathed *and was sold to a Canadian company.* | | |

Table 7: The transformation rule pattern for disembedding coordinate verb phrases. A framed pattern represents the part of the input that is extracted and rephrased into a self-contained sentence. The underlined pattern refers to its referent.

<table>
<tr><th>Rule</th><th>Tregex pattern</th><th>Extracted sentence</th></tr>
<tr><td>PostListNP-Extractor</td><td>ROOT <<: (S < (NP $.. (VP << (NP=np1 < (NP $.. NP=np2))));<br>Extraction ← replace np1 with np2</td><td>Extraction.</td></tr>
<tr><td colspan="3">Example: Demola Aladekomo is a computer engineer, technology pioneer, entrepreneur and philanthropist.</td></tr>
<tr><td>PreListNP-Extractor</td><td>ROOT <<: (S < (NP=np1 < (NP $.. NP=np2) $.. VP));<br>Extraction ← replace np1 with np2</td><td>Extraction.</td></tr>
<tr><td colspan="3">Example: At the end of the progression, 47 seconds into the song, the intensity of the synthesizer rises before an organ, bass guitar, and piano subsequently enter.</td></tr>
</table>

Table 8: A selection of transformation rule patterns. A framed pattern represents the extracted part of a sentence. An underlined pattern refers to its referent. A pattern in bold will be excluded in the remaining sentence.

<table>
<tr><th>Rule</th><th>Tregex pattern</th><th>Extracted sentence</th></tr>
<tr><td>Non-Restrictive-Apposition-Extractor</td><td>ROOT <<: (S < (VP < +(VP) /.*/) & << (NP $+ (/,/ $+ (NP !$ CC & ?$+ /,/))))</td><td>Attachment phrase + Be + extraction.</td></tr>
<tr><td colspan="3">Example: The president of Lithuania, Antanas Smetona, proposes armed resistance.</td></tr>
<tr><td>Restrictive-Apposition-Extractor</td><td>(((PRP\\$|DT)\\ s)*(JJ\\ s)*((NN|NNS|NNP|NNPS)\\ s))+((CC\\ s)((PRP\\$|DT)\\ s)*(JJ\\ s)*((NN|NNS|NNP|NNPS)\\ s)) followed by a named entity</td><td>Attachment phrase + Be + extraction.</td></tr>
<tr><td colspan="3">Example:</td></tr>
</table>

Table 9: A selection of transformation rule patterns. A framed pattern represents the extracted part of a sentence. An underlined pattern refers to its referent. A pattern in bold will be excluded in the remaining sentence.

<table>
<tr><th>Rule</th><th>Tregex pattern</th><th>Extracted sentence</th></tr>
<tr><td>Prepositional-Attachedto-VPExtractor</td><td>ROOT <<: (S < +(S|VP) (VP < (PP $– NP|PP)) & < VP)</td><td><u>This</u> + Be + PP.</td></tr>
<tr><td colspan="3">Example:</td></tr>
<tr><td>Prepositional-Initial-Extractor</td><td>ROOT <<: (S <, (PP ?$+ /,/ & $ + + VP))</td><td><u>This</u> + Be + PP.</td></tr>
<tr><td colspan="3">Example: After his retirement, he took charge as director of the Indian Institute of Science, holding this position from 1915 to 1921.</td></tr>
<tr><td>Prepositional-MiddleFinal-Extractor</td><td>ROOT <<: (S < VP & << (/,/ $+ (PP ?$+ /,/)))</td><td><u>This</u> + Be + PP.</td></tr>
<tr><td colspan="3">Example: Founded as a Numidian colony in 4th to 5th century BC, it later became a Roman town in the province of Africa, before its eventual abandonment around 9th to 10th century.</td></tr>
</table>

Table 10: A selection of transformation rule patterns. A framed pattern represents the extracted part of a sentence. An underlined pattern refers to its referent. A pattern in bold will be excluded in the remaining sentence.

<table>
<tr><th>Rule</th><th>Tregex pattern</th><th>Extracted sentence</th></tr>
<tr><td>Adjectival-Adverbial-Initial-Extractor</td><td>ROOT <<: (S <, (ADJP|ADVP $+ (/,/ $ + + VP)))</td><td><u>This</u> + Be + ADJP|ADVP.</td></tr>
<tr><td colspan="3">Example: Meanwhile, unemployment in France threw skilled workers down to the level of the proletariat.</td></tr>
<tr><td>Adjectival-Adverbial-Middle-Final-Extractor</td><td>ROOT <<: (S < VP & << (/,/ $+ (ADJP|ADVP ?$+ /,/)))</td><td><u>This</u> + Be + ADJP|ADVP.</td></tr>
<tr><td colspan="3">Example: Gustafsson lived a normal life until 2004, almost 44 years after the event.</td></tr>
</table>

Table 11: A selection of transformation rule patterns. A framed pattern represents the extracted part of a sentence. An underlined pattern refers to its referent. A pattern in bold will be excluded in the remaining sentence.

| Rule | Tregex pattern | Extracted sentence |
| --- | --- | --- |
| LeadNPExtractor | ROOT <<: (S <, (NP $+ **(/,/** $+ NP & $ + + VP))) | *This* + Be + NP. |
| Example: *Six days later,* NATO took over leadership of the effort. | | |

Table 12: The transformation rule pattern for splitting and rephrasing leading noun phrases. A framed pattern represents the part of the input that is extracted and turned into a stand-alone sentence. A pattern in bold will be deleted from the remaining sentence.
