# Supplementary material for: Reading bots: The implication of deep learning on guided reading
Source: Front Psychol. 2023 Feb 6;14:980523. doi: 10.3389/fpsyg.2023.980523 (PMC9939479; doi:10.3389/fpsyg.2023.980523)
Supplement: Supplementary file 2 [file Data_Sheet_1.ZIP › readingbots-frontend.zip!/transmanager-readingbots-frontend-/src/pages/document.ejs]

Reading Bots


Hi there! Please

**enable Javascript**

in your browser to use Reading Bots!
